# GIF1 controls ear inflorescence architecture and floral development by regulating key genes in hormone biosynthesis and meristem determinacy in maize

**DOI:** 10.1186/s12870-022-03517-9

**Published:** 2022-03-18

**Authors:** Manfei Li, Yuanyuan Zheng, Di Cui, Yanfang Du, Dan Zhang, Wei Sun, Hewei Du, Zuxin Zhang

**Affiliations:** 1 College of Life Science, Yangtze University, Jingzhou, 434025 People’s Republic of China; 2 National Key Laboratory of Crop Genetic Improvement, Hubei Hongshan Laboratory, Huazhong Agricultural University, Wuhan, 430070 People’s Republic of China; 3 College of Agronomy, Tarim University, Alar, Xinjiang 843300 People’s Republic of China

**Keywords:** Spikelet pair meristem, Branch meristem, Carpel, *Clavata3*/*esr*-related gene, Inflorescence

## Abstract

**Background:**

Inflorescence architecture and floral development in flowering plants are determined by genetic control of meristem identity, determinacy, and maintenance. The ear inflorescence meristem in maize (*Zea mays*) initiates short branch meristems called spikelet pair meristems, thus unlike the tassel inflorescence, the ears lack long branches. Maize growth-regulating factor (GRF)-interacting factor1 (GIF1) regulates branching and size of meristems in the tassel inflorescence by binding to *Unbranched3*. However, the regulatory pathway of *gif1* in ear meristems is relatively unknown.

**Result:**

In this study, we found that loss-of-function *gif1* mutants had highly branched ears, and these extra branches repeatedly produce more branches and florets with unfused carpels and an indeterminate floral apex. In addition, GIF1 interacted in vivo with nine GRFs, subunits of the SWI/SNF chromatin-remodeling complex, and hormone biosynthesis-related proteins. Furthermore, key meristem-determinacy gene *RAMOSA2* (*RA2*) and CLAVATA signaling-related gene *CLV3/ENDOSPERM SURROUNDING REGION* (*ESR*) *4a* (*CLE4a*) were directly bound and regulated by GIF1 in the ear inflorescence.

**Conclusions:**

Our findings suggest that GIF1 working together with GRFs recruits SWI/SNF chromatin-remodeling ATPases to influence DNA accessibility in the regions that contain genes involved in hormone biosynthesis, meristem identity and determinacy, thus driving the fate of axillary meristems and floral organ primordia in the ear-inflorescence of maize.

**Supplementary Information:**

The online version contains supplementary material available at 10.1186/s12870-022-03517-9.

## Background

Maize (*Zea mays* L.), one of the most widely cultivated crop plants in the world, produces two distinct inflorescences, in contrast to other related grasses such as *Sorghum bicolor* and *Oryza sativa*, each of which has a panicle of perfect flowers. Whereas the maize male inflorescence (tassel) is a panicle with multiple long branches producing a variable number of spikelets, the female inflorescence (ear) is covered with short branches (*i.e.* spikelet pair branches) and in each spikelet is two florets, the lower of which aborts. The maize ear, with its dozens to hundreds of kernels, is an important reproductive and agronomic tissue. In normal development of the ear, the inflorescence meristem (IM) first initiates indeterminate spikelet pair meristems (SPMs), which are short branches [1, 2]. Thus, unlike tassels, ears lack long branches. The ear florets initiate a palea, a lemma, two lodicules, three stamens, and three carpels. After initiation, the stamens abort in female flowers, but the carpels develop into a single pistil by fusing congenitally along their edges; two indeterminate abaxial carpels fuse to form the silk, and the third elongates to cover the ovule forming the ovary wall [3, 4]. After double fertilization, ovules enclosed by carpels develop into kernels (caryopses). Therefore, branching and gynoecium development are both critically important for inflorescence architecture and floral fertility, as well as for grain yield.

Previous studies revealed a complex functional hierarchy of genes involved in inflorescence branching in maize. Mutations in three classical *RAMOSA* (*RA*) genes produce highly branched male and female inflorescences, in which the SPMs are converted into branch meristems (BMs) [5]. The *RA3* encodes a trehalose-6-phosphate phosphatase [6], which removes the phosphate from trehalose 6-phosphate (T6P) to produce free trehalose, suggesting that sugar signaling triggered by polysaccharides may be involved in inflorescence architecture [7–9]. Mutation of *RA3* leads to reduced expression of the zinc-finger domain protein-encoding gene *RA1* [10], suggesting that *RA3* may regulate *RA1* directly or indirectly. The *RA2* encodes a lateral organ boundary (LOB) domain transcription factor (TF) required for initiation of axillary meristems in both inflorescences. In *ra2* mutants, expression of *RA1* is also down-regulated, suggesting that both *RA2* and *RA3* positively regulate expression of *RA1* [11]. Genes in the CLAVATA-WUSCHEL (CLV-WUS) feedback loop, such as *Fasciated ear2* (*FEA2*) [12], *FEA3* [13] and *Thick tassel dwarf1* (*TD1*) [14] also regulate inflorescence branching.

Growth-regulating factor (GRF)-interacting factor1 (GIF1), which is one of the transcriptional coactivators of plant-specific growth-regulating factors (GRFs), has been characterized as a major regulator of plant vegetative and reproductive development [15–20]. In Arabidopsis (*Arabidopsis thaliana*), *GIF1/AN3* is required for maintenance of the shoot apical meristem; establishment of the carpel margin meristem; development of flower organs, cotyledons, and leaves; cell proliferation and expansion; and lateral organ growth [17, 21–25]. In rice, *OsGIF1* positively regulates plant height, leaf size, stem internodes, grain size, number of grains per panicle, and branches per panicle [26, 27], playing a major role in regulating the size of vegetative and reproductive organs and the number of inflorescence branches.

Recently, we found that maize *gif1* mutants have fewer branches in the tassels than wild-type plants but extra branches in the ears [28], showing opposite effects of *GIF1* on the branches in the tassel and the ear. Here, we compared the detailed morphological and anatomical differences in the ear inflorescence between the *gif1-1* mutant and the wild type. We further identified GIF1-interacting proteins using immunoprecipitation-mass spectrometry (IP-MS) and GIF1 target genes through chromatin immunoprecipitation sequencing (ChIP-seq), integrating these data with transcriptome data. We propose that GIF1 regulates axillary meristems and floral organ primordia in the ear-inflorescence by targeting genes in hormone biosynthesis, genes in meristem identity and determinacy involving *RAMOSA* and *CLV-WUS* pathway.

## Materials and methods

### Plant materials and phenotypic characterization

The maize *gif1-1* mutant was from our lab, which was originally found in the BS238 family line. The *gif1-1* mutant and *GIF1-GFP* overexpressing lines were planted at Wuhan (30°N, 114°E), China. The phenotypes of traits including plant height, ear height and leaf length. The sample size for each phenotypic value was more than 30 individuals. All methods, including plant experimental research, were performed in accordance with the relevant institutional, national, and international guidelines and legislation.

### *GIF1-GFP* fusion construct and genetic transformation

The *GIF1* coding sequence was amplified with primers *GIF1-F* and *GIF1-R* (Supplemental Table 2) and fused to the *green fluorescent protein* (*GFP*) coding sequence. The *GIF1-GFP* fusion construct (Supplemental Fig. 4) was cloned into the pZZ01523 vector (Life Science and technology Center, China National Seed Group CO., LTD, China. http://www.chinaseeds-lstc.com), and the resulting vector was transformed into the maize ZZC01 line by *Agrobacterium*-mediated transformation [29]. Transgenic genotypes were determined using *Trans-F/R* primers, with the Trans-F primer designed against the *GIF1* sequence and the *Trans-R* primer designed against the vector sequence. The expression level of *gif1* was measured using *Q-gif1-F/R* primers designed against the 3ʹ-UTR of *gif1* (Supplemental Table 3).

### Genetic complementation

To analyze biological functions, phenotypes of *GIF1-GFP* overexpressing lines were evaluated. Additionally, the *gif1* allele of the *gif1-1* mutant was introduced into ZZC01 genetic background through two cycles of backcrossing using marker-assisted selection. Those + /*gif1* heterozygotes were crossed to line OE2. Individuals with *gif1gif1* genotype and overexpressing *GIF1-GFP* (referred to as complemented plants) were selected through genotyping using gene-specific primers (Supplemental Table 3) and evaluated for phenotypic rescue of plant characteristics and inflorescence architecture.

### Microscopy observation

For stereomicroscope observation, immature ears were collected from the *gif1-1* mutant and its wild-type siblings, and the complemented plants. The ear inflorescence images were acquired using a Nikon SMZ25 microscope (Nikon, Japan) and merged by NIS (Nikon Imaging Software) elements. Immature ear inflorescences (5 mm) from the *gif1-1* mutant and wild type were sampled according to Li et al*.* [30]. A sequential sampling procedure was performed from the beginning of the ninth leaf stage to observe the time course of inflorescence development. Inflorescence samples were fixed in a glutaraldehyde solution (2.5% v/v glutaraldehyde in 0.08 M phosphoric acid buffer) for 24 h at 4 °C and then dehydrated through a graded series of ethanol from 30 to 90%. Tissue samples were dried using a critical point dryer, sputter coated with gold palladium for 45 s, and observed on a Hitachi S-4700 scanning electron microscope (Hitachi, Japan) at an accelerating voltage of 5 kV [28].

### Immunoprecipitation-mass spectrometry (IP-MS)

Developing ears ~ 5 mm in length were collected from line OE2 and ground in a mortar using liquid nitrogen. The frozen powder was mixed with ice-cold extraction buffer (50 mM Tris–HCl, 150 mM NaCl, 1 mM phenylmethanesulfonyl fluoride, and 1% Triton X-100). The mixture was centrifuged at 3,000 g for 15 min. The supernatant was used as total protein for immunoblotting. Total proteins were separated using 30% sodium dodecyl sulfate–polyacrylamide gel electrophoresis (SDS-PAGE). The gel was stained with 0.2% (w/v) silver nitrate. For immunoblotting, proteins were electrotransferred onto polyvinylidene difluoride membranes under 100 V and 60 mA for 1 h. A 1:2,000 dilution of anti-GFP mouse monoclonal antibody (M048-3, MBL, China) was used as the primary antiserum, followed by incubation with a 1:3,000 horseradish peroxidase (HRP)-goat anti-rabbit secondary antibody (ab6721, Abcam).

For co-immunoprecipitation assay, total proteins were placed on ice for 30 min and centrifuged at 10,000 g for 10 min at 4 °C. GFP-Trap®_MA beads (ChromoTek, Planegg-Martinsried, Germany) were washed twice with 500 µL of extraction buffer. Each sample was mixed with 25 µL of clean beads, and the mixture was tumbled end-over-end for 3 h at 4 °C. The beads were magnetically separated, washed twice, and then heated in 100 µL of 2 × SDS sample buffer at 95 °C. Proteins from corresponding non-transgenic line of line OE2 were used as a negative control. Immunocomplexes were analyzed by mass spectrometry. The IP-MS experiment was performed with three biological replicates. Proteins identified in at least two IP-MS experiments were referred to as GIF1-interacting proteins.

### Firefly luciferase complementation imaging (LCI) assay

The open reading frames (ORFs) of *GIF1* and the four genes encoding GIF1-interacting proteins were separately cloned into both JW771 (NLUC) and JW772 (CLUC) [31] using a ClonExpress II One Step Cloning Kit (Vazyme Biotech, Nanjing, China). The constructs were transformed into *Agrobacterium tumefaciens* strain GV3101. The transformed *Agrobacterium* cells were grown to OD(optical density)_600_ = 0.8, pelleted, and resuspended in infiltration buffer (10 mM methylester sulfonate, 10 mM MgCl_2_, and 150 mM acetosyringone, pH 5.7) and then infiltrated in different combinations into 3-week-old *N. benthamiana* leaves using a needleless syringe. After 48 h under 16 h of light and 8 h of dark, leaves were injected with 1 mM luciferin (Promega, Madison, WI, USA). The resulting luciferase signals were observed using a Tanon-5200 image system (Tanon Science, Shanghai,, China). The LCI assay was performed three times independently.

### Transcriptome library construction and sequencing

The transcriptome library construction and RNA-seq analysis were done as Zhang et al. [28]. Ten immature ears (~ 5 mm) from the *gif1-1* and wild-type sibling were collected and pooled, respectively, with three biological replicates. Fresh immature ears were immediately frozen in liquid nitrogen. Total RNA was extracted from each pool using Trizol (Life Technologies, Invitrogen, USA). After removing DNA with RQ1DNase (Promega, USA), 10 mg of total RNA was used for RNA-seq library preparation. Polyadenylated mRNAs were purified and concentrated with oligo (dT)-conjugated magnetic beads (Life Technologies, USA). Purified mRNAs were fragmented at 95 °C for 1 min, followed by end repair and 5′-adaptor ligation. Reverse transcription was then performed with a specific primer harboring a 3′-adaptor sequence and randomized hexamer. The cDNA was purified and amplified using random hexamers, and PCR products of 200 to 500 bp were collected, purified, quantified, and subjected to paired-end sequencing on an Illumina HiSeq 2000 system (Illumina Inc., San Diego, CA, USA) at the Beijing Genomics Institute (BGI).

For quantifying gene expression level, clean reads were mapped to the maize reference genome (B73 RefGen_v4) using SOAPaligner/SOAP2 [32] with no more than five mismatches allowed in the alignment. Gene expression level was calculated using the FPKM method (fragments per kilobase transcriptome per million mapped). Differentially expressed genes (DEGs) between the *gif1-1* mutant and the wild type were identified using *p* < 10^–5^ and a two-fold difference. The DEGs are listed in Supplemental Data Set 2. Gene Ontology (GO) analysis (by agriGO, http://systemsbiology.cau.edu.cn/agriGOv2/) was used to identify the enrichment of the DEGs.

### Chromatin immunoprecipitation (ChIP)-sequencing and data analysis

The ChIP-Seq was done as Zhang et al. [28]. Approximately 1 g of ear inflorescences (~ 5 mm) was harvested from *p35S::GIF1-GFP* line OE2 grown in a greenhouse with three biological replicates. Expression of the fused GIF1-GFP was verified by protein gel blotting using anti-GFP antibody (Abcam, AB290) at a dilution of 1:1,000 (v/v) in Tris-buffered saline buffer containing 5% nonfat milk powder. The inflorescences were immediately cross-linked in buffer containing 1% (v/v) formaldehyde for 15 min under vacuum, followed by addition of glycine to a concentration of 0.1 M and infiltration for 5 min. After three washes with distilled water (4 °C), the cross-linked tissues were dried with paper towels and flash-frozen in liquid nitrogen. Frozen tissues were ground thoroughly to a fine powder, which was then transferred to a precooled 50-mL tube with 20 mL of cold complete extraction buffer 1 (0.4 M sucrose, 10 mM Tris–HCl, pH 8.0, 10 mM MgCl, 2.5 mM β-mercaptoethanol, and Plant Protease Inhibitor Cocktail (P9599, Sigma-Aldrich). Homogenized tissues were centrifuged for 20 min at 1,000 g at 4 °C. The pellets were washed five times with 5 mL of complete extraction buffer 2 (0.25 M sucrose, 10 mM Tris–HCl, pH 8.0, 10 mM MgCl_2_, 1% (v/v) Triton X-100, 5 mM β-mercaptoethanol, and Plant Protease Inhibitor Cocktail) and once with extraction buffer 3 (1.7 M sucrose, 10 mM Tris–HCl, pH 8.0, 2 mM MgCl_2_, 0.15% (v/v) Triton X-100, 5 mM β-mercaptoethanol, and Plant Protease Inhibitor Cocktail). Washed pellets were resuspended in 300 mL of sonication buffer (50 mM Tris–HCl, pH 8.0, 10 mM EDTA, 1% SDS, and Plant Protease Inhibitor Cocktail), and the suspension was treated with a Bioruptor (Diagenode, Belgium) for 8 to 10 cycles with settings 30 s ON/30 s OFF at 4 °C. The sonicated sample was centrifuged for 10 min at 12,000 g at 4 °C, and the supernatant was collected and used for chromatin isolation. The extracted chromatin was immunoprecipitated with anti-GFP antibody (Invitrogen, A11122) with a Plant ChIP-seq kit (Diagenode, Belgium) according to the manufacturer’s instructions. Following de-cross-linking, isolation, and purification of the immunoprecipitated DNA, libraries were constructed using an Ovation Low Input DR kit (NuGEN Technologies, San Carlos, CA, USA). Two input and two IP libraries were subjected to sequencing on an Illumina HiSeq 2000 sequencer (Illumina Inc., USA).

ChIP-seq reads were aligned to the maize reference genome (AGPv4) using Hisat2 v.2.0.5 [33]. Only uniquely mapped reads were considered for further processing. PCR duplicates were removed using Picard Mark Duplicates (v.2.9.0; http://picard.sourceforge.net/). Peak calling was performed with MACS (v.1.4.2) [34]. Peaks were identified as significantly enriched (*p* < 10^–5^) in each of the ChIP-seq libraries compared with input DNA. The FGS (Functional Gene Set) gene model within 10 kb of the peak summit was considered as a putative target of GIF1. ChIP tracks showing GIF1-GFP fusion protein binding sites were visualized using integrative genomics viewer [35].

### ChIP-qPCR

The ChIP-qPCR was done as Zhang et al. [28]. To detect specific DNA targets, ChIP-qPCR was performed to quantify DNA targets immunoprecipitated by anti-GFP antibody relative to input DNA using SYBR Green qPCR Master Mix (Bio-Rad, Hercules, CA, USA) with three biological replicates, each with three technical replicates. The DNA target-specific primers used for the ChIP-PCR assay are listed in Supplemental Table 3. The abundance of a target was normalized to that of nonspecific genomic regions, and fold enrichment of the DNA target relative to the input sample was then calculated. Significant differences were estimated by a Student’s t-test.

### Reverse-transcription quantitative PCR (RT-qPCR)

To analyze gene expression, immature ears (~ 5 mm) were collected from the *gif1-1* and wild-type plants. Total RNA was extracted from plant tissues using Ambion Pure Link Plant RNA Reagent (Life Technologies, USA) and reverse-transcribed with M-MLV reverse transcriptase (Life Technologies, USA) according to the manufacturer’s instructions. RT-qPCR was performed using a SYBR Green qRT-PCR kit (Bio-Rad, USA) according to the manufacturer’s instructions with three biological replicates; each replicate contained 10 individuals. Fold changes in RNA transcripts were calculated by the 2^−ΔCt^ method with maize *Actin* gene (Zm00001d010159) as an internal control. All reactions were performed on a CFX96 real-time system (Bio-Rad). All primers used for RT-qPCR are listed in Supplemental Table 3.

## Results

### The *gif1* mutant has highly branched ears with unfused carpels 

**Fig. 1:**
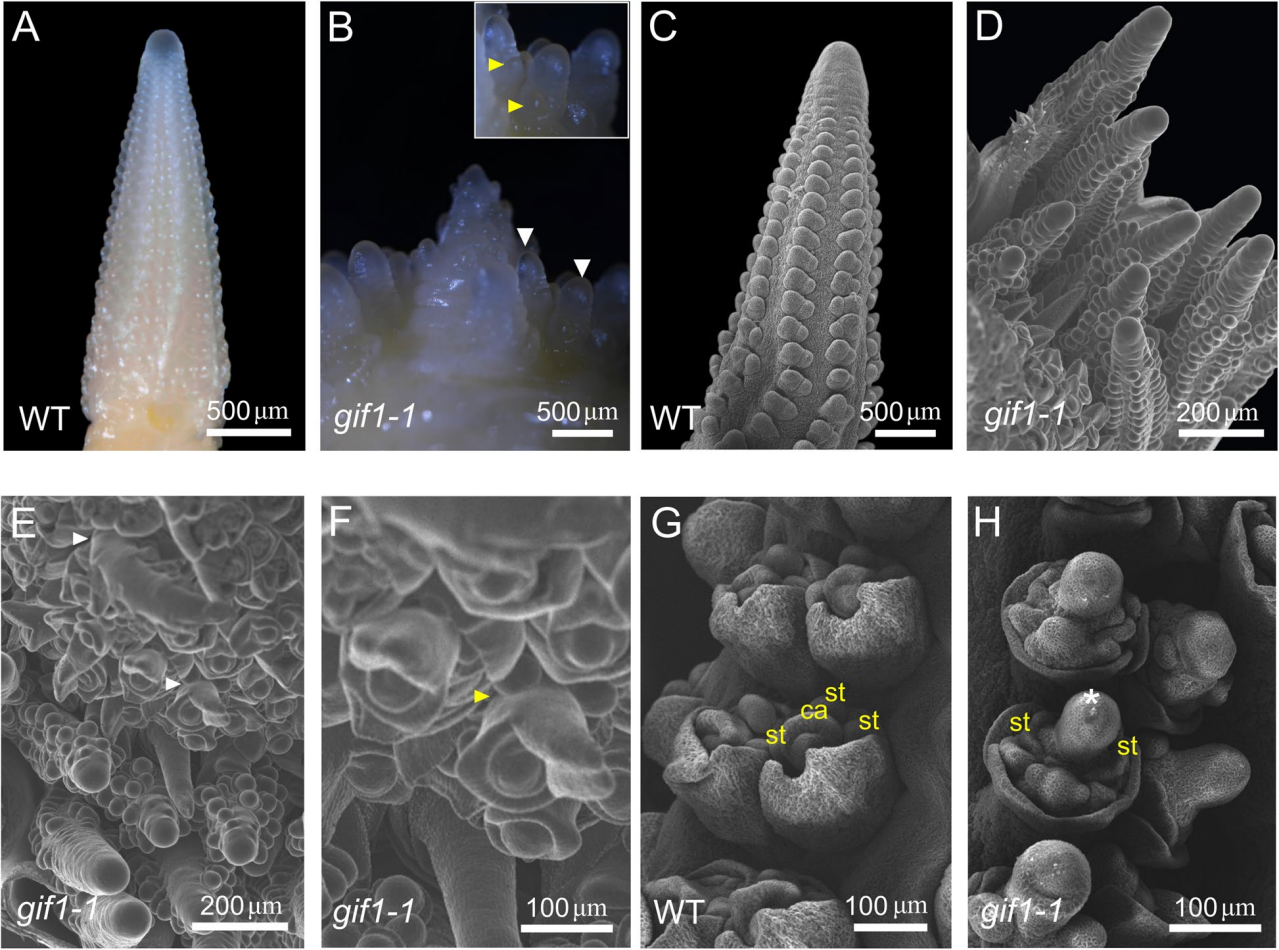
Obvious morphological and anatomical differences between the *gif1* mutant and wild-type sibling in the inflorescence architecture and floral organs. **A** Developing inflorescence architecture of wild type. **B** Highly branched ear and branch differentiating axillary meristems (zoom) in the *gif1*. White arrows point to axillary meristems, yellow arrows point to the spikelet meristems on the branch. **C** Scanning electron microscopy of developing inflorescence architecture in wild type. **D** Scanning electron microscopy of branch differentiating axillary meristems in the *gif1*. **E** and **F** Scanning electron microscopy of branches converted from *gif1* florets (**E**) and their enlarged images (**F**). Arrows point to branches converted from florets. G Scanning electron microscopy of the wild-type floret. **H** Scanning electron microscopy of the *gif1-1* mutant. Asterisk points to the naked ovule primordia. WT, wild type. ca, carpel primordium; st, stamen primordium

To uncover the roles of *GIF1* in the ear-inflorescence development, we observed the initiation and differentiation of meristems during inflorescence development. We found that wild-type maize ears produced paired spikelets with no long branches (Fig. 1A and C). By contrast, the ears of *gif1-1* mutants frequently displayed highly short branched inflorescences (Fig. 1B and D). Spikelet meristems (SMs) on the branched inflorescences produced variable numbers of BMs or SPMs (Fig. 1B and D) and floral meristems (FMs) on the branched inflorescence also convert into BMs (Fig. 1E and F), indicating that *GIF1* regulates determinacy of BMs and SMs or FMs identity.

In wild-type florets, development of the three stamen primordia is arrested and carpel primordia fuse to make a functional silk, and the ovule is well enclosed by the carpel (Fig. 1G). In *gif1-1* florets, development of stamen primordia was also arrested; however, carpel primordia frequently failed to be initiated, or were initiated but failed to fuse, or were well developed in only one of the two florets (Fig. 1H). The ovule primordia were enlarged and naked (Fig. 1H), and the floral apex was indeterminate and frequently initiated extra ovule-like protrusions in *gif1-1* florets, showing similarities to the *ifa1* mutant [36], such as an expanded nucellus. These results indicated that *GIF1* regulates fate and determinacy of meristems on the ear inflorescence.

### Overexpression of *GIF1* rescues the defective phenotypes of the *gif1* mutant

**Fig. 2:**
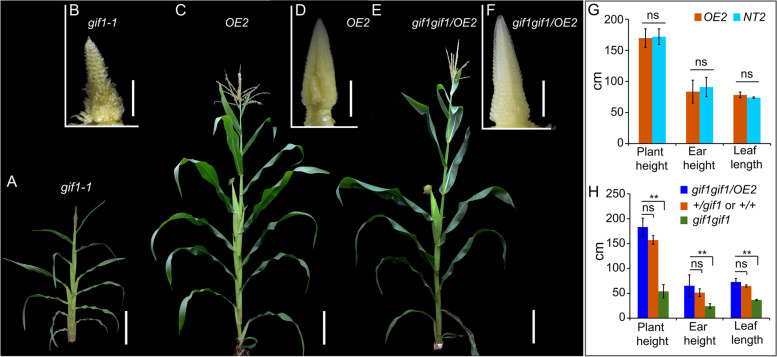
Overexpressing *GIF1-GFP* rescues the defective phenotypes on the ear inflorescence of the *gif1* mutant under ZZC01 background. **A** and **B** Whole plant (**A**) and ear inflorescence (**B**) of the *gif1-1* mutant. The *gif1-1* mutant produced highly branched inflorescence (**B**). **C** and **D** Whole plant (**C**) and ear inflorescence (**D**) of transgenic line OE2 overexpressing *GIF1-GFP*. **E** and **F** Whole plant (**E**) and ear inflorescence (**F**) of the complemented line. Gene-specific molecular markers were used to genotype the *gif1gif1*/OE2 individuals in the progeny families derived from + /*gif1* (ZZC01) × *GIF1-GFP*-OE2 (ZZC01), which are homozygous genotype at the *gif1* locus and are expressing the GIF1-GFP. **G** Plant height, ear height and leaf length of transgenic line OE2 overexpressing *GIF1-GFP* and corresponding non-transgenic line (NT) under ZZC01 background. **H** Plant height, ear height and leaf length of complementation individuals with *gif1gif1/OE2*, wild-type individuals with *GIF1/gif1* or *GIF1/GIF1* genotype, and *gif1gif1* individuals identified from the progeny families derived from + */gif1*(ZZC01) × *GIF1-GFP*-OE2 (ZZC01). The scale bars = 500 μm in (**B**), (**D**) and (**F**), and 10 cm in (**A**), (**C**) and (**E**). Sample size = 30 in (**G**) and (**H**). The column shows mean ± standard deviation (s.d.), the statistical significance was estimated using a Student’s t-test. ** *P* < 0.01. ns: not significant statistically

To uncover the function of *GIF1* in the ear inflorescence, we created five transgenic lines by introducing the *Ubi* promoter-driven GIF1-GFP constructs into the maize ZZC01 inbred line. The *gif1-1* mutant appeared as a dwarf plant (Fig. 2A), while transgenic lines overexpressing *GIF1-GFP* (OE) showed normal characteristics with greater plant height and ear height, and longer leaves than the *gif1-1* mutant (Fig. 2C, G, H), but displayed non-significant difference from its non-transgenic sibling (NT2) (Fig. 2G). The ears from overexpression line (OE2) generated orderly and fertile florets without long branches (Fig. 2D). As contrast, *gif1* ears produced long branches at the base of the ear inflorescence (Fig. 1D and Fig. 2B). To reveal the roles of *GIF1* in the ear development, we first introduced *gif1* into ZZC01 genetic background through two generations of backcrossing, and then crossed transgenic line OE2 to + */gif1* heterozygotes under ZZC01 genetic background followed by one generation of selfing. Those plants with homozygous *gif1*/*gif1* and overexpressing *GIF1-GFP* which are referred to as complementation individuals were then selected by genotyping. All of these complementation individuals had normal plant characteristics and inflorescence architectures, both female and male florets were well developed and fertile (Fig. 2E, F). In addition, plant height, ear height and leaf length of complementation individuals were slightly greater than that of wild-type individuals with + */gif1* or + */* + genotype although the difference is not statistically significant (Fig. 2H), but were significantly different from in the defective phenotypes of those *gif1gif1* individuals (*n* = 30). The results show that overexpression of *GIF1-GFP* can rescue the defective phenotypes on the ear inflorescence of the *gif1* mutant.

### GIF1 interacting proteins are involved in diverse biological processes

To identify GIF1-interacting proteins, we performed an IP assay using anti-GFP antibody in 5-mm ears from transgenic line OE2 with three biological replicates (Supplemental Fig. 1). We identified 56 GIF1-interacting proteins substantially enriched in at least two biological replicates (Table 1). Some of these proteins might indirectly interact with GIF1, given that GIF1 is a coactivator of GRFs; regardless, they are likely to be components of a GIF1-GRF recruiting complex. Consistent with proteins identified previously in Arabidopsis [37] and maize, nine GRFs, one SWI3D, two SNF12s, two Agenet domain proteins, two helicases, two actin-related proteins (ARP4 and ARP7), and one ATPase were identified as GIF1-interacting proteins in developing ears (Table 1 and Supplemental Table 1). These proteins were identified as subunits of the SWI/SNF complex that regulates chromatin structure by altering nucleosome composition and interactions [38]. And these proteins have been repeatedly identified to interact with GIF1 in different plant species, suggesting a reliable interaction between GIF1 and SWI/SNF subunits.

**Table 1:** GIF1-interacting proteins identified in maize ears by Co-IP

Gene ID	Function or domain	No. of unique peptides			Annotation	Expression at the ear (FPKM)
		Exp1	Exp2	Exp3		
Zm00001d027326	ARP	8	4	9	Actin related protein 4	58.73
Zm00001d013410		10	10	3	Actin related protein 1	133.24
Zm00001d053177		11	10	2	Actin related protein 7	155.76
Zm00001d011087		13	10	4	Actin related protein 7	195.84
Zm00001d047079	Agenet domain	18	10	3	G2428-1	8.58
Zm00001d029889		22	14	2	G2428-1	12.25
Zm00001d051840	Helicase	4	4	0	RNA helicase 2	48.5
Zm00001d044540		6	3	9	DNA replication licensing factor MCM4	42.79
Zm00001d017742	GRF	1	1	1	GRF1	174.02
Zm00001d021362		1	1	1	GRF10	57.97
Zm00001d000238		1	2	1	GRF11	235.59
Zm00001d045533		1	1	1	GRF12	207.44
Zm00001d033876		1	1	1	GRF15	152.36
Zm00001d051456		2	2	1	GRF17	110.11
Zm00001d006348		1	1	1	GRF4	61.03
Zm00001d037117		1	1	1	GRF7	29.60
Zm00001d017602		1	1	1	GRF18	172.67
Zm00001d022322	SWI/SNF subunit	4	3	0	SWI/SNF component SNF12 homolog	17.40
Zm00001d007039		5	5	4	SWI/SNF component SNF12 homolog	25.78
Zm00001d013391		3	3	0	SWI/SNF complex subunit SWI3D	9.16
Zm00001d012052	RRM/RBD/RNP	2	2	0	RRM/RBD/RNP motifs protein	372.4
Zm00001d002244		3	3	2	RNA recognition motif containing protein	82.37
Zm00001d011336	hnRNP	5	4	2	Heterogeneous nuclear ribonucleoprotein A3	80.57
Zm00001d041214	ATPase	9	3	0	ATPase 4 plasma membrane type	57.64
Zm00001d038923	Small GTPase	6	6	1	Guanine nucleotide-binding protein β subunit	327.87
Zm00001d034590		3	5	0	Rho GTPase 1 mitochondrial	34.43
Zm00001d017239		4	3	0	Ran GTPase-activating protein 2	36.21
Zm00001d037004		4	4	2	Ras-related protein RABA1d	77.91
Zm00001d009572		3	4	1	Ras-related protein RABA1f	48.05
Zm00001d011855		3	5	2	Arf-GTPase-activating protein AGD11	67.93
Zm00001d022316	Protein biosynthesis and metabolism	4	2	0	Proteasome subunit alpha type	135.42
Zm00001d027896		4	4	3	Proteasome subunit alpha type	137.84
Zm00001d049230		2	3	3	SKP1-like protein 1A	880.84
Zm00001d046449		13	8	0	Elongation factor 1-alpha9	615.43
Zm00001d034856		6	6	2	Elongation factor TU	89.24
Zm00001d053196		3	5	5	Proline-tRNA ligase cytoplasmic	33.85
Zm00001d048021	Hormone-related	3	3	0	Allene oxide synthase1 (AOS1)	44.48
Zm00001d014887		4	3	3	Nana Plant2 (NA2)	215.56
Zm00001d041711		0	1	1	Auxin-binding protein 1(Abp1)	325.99
Zm00001d037700	Chaperone	9	17	11	Heat shock protein 4	41.36
Zm00001d017401		4	7	3	Hsp70-Hsp90 organizing protein 3	32.23
Zm00001d032789		9	15	2	Chaperonin2	41.26
Zm00001d050375	Regulatory molecule	14	13	4	14–3-3-like protein	410.94
Zm00001d052698		8	9	0	14–3-3-like protein GF14 nu	37.90
Zm00001d003401		9	12	2	14–3-3-like protein GF14-6	550.72
Zm00001d030968	Nucleus	0	1	4	Flowering locus K homology domain	96.52
Zm00001d042091	Developmental process	1	0	4	Flowering time control protein FY	13.25
Zm00001d024523		2	2	0	Ramosa1 enhancer locus2	87.75
Zm00001d009850	Intracellular transport	5	3	2	Importin subunit alpha	86.48
Zm00001d033734		6	6	3	Coatomer subunit alpha-1	10.78
Zm00001d007758		8	2	2	Coatomer subunit gamma	19.90
Zm00001d049155		2	1	1	Sec23/Sec24 protein	28.91
Zm00001d028143		1	1	0	COP9 signalosome complex subunit 4	62.60
Zm00001d032859	Cell division	13	7	1	Cell division control protein 48 homolog	36.74
Zm00001d014124		13	7	1	Cell division cycle protein 48	50.72
Zm00001d040429	Argonaute	2	7	1	Argonaute105	74.68

*EXP* Experiment

**Fig. 3:**
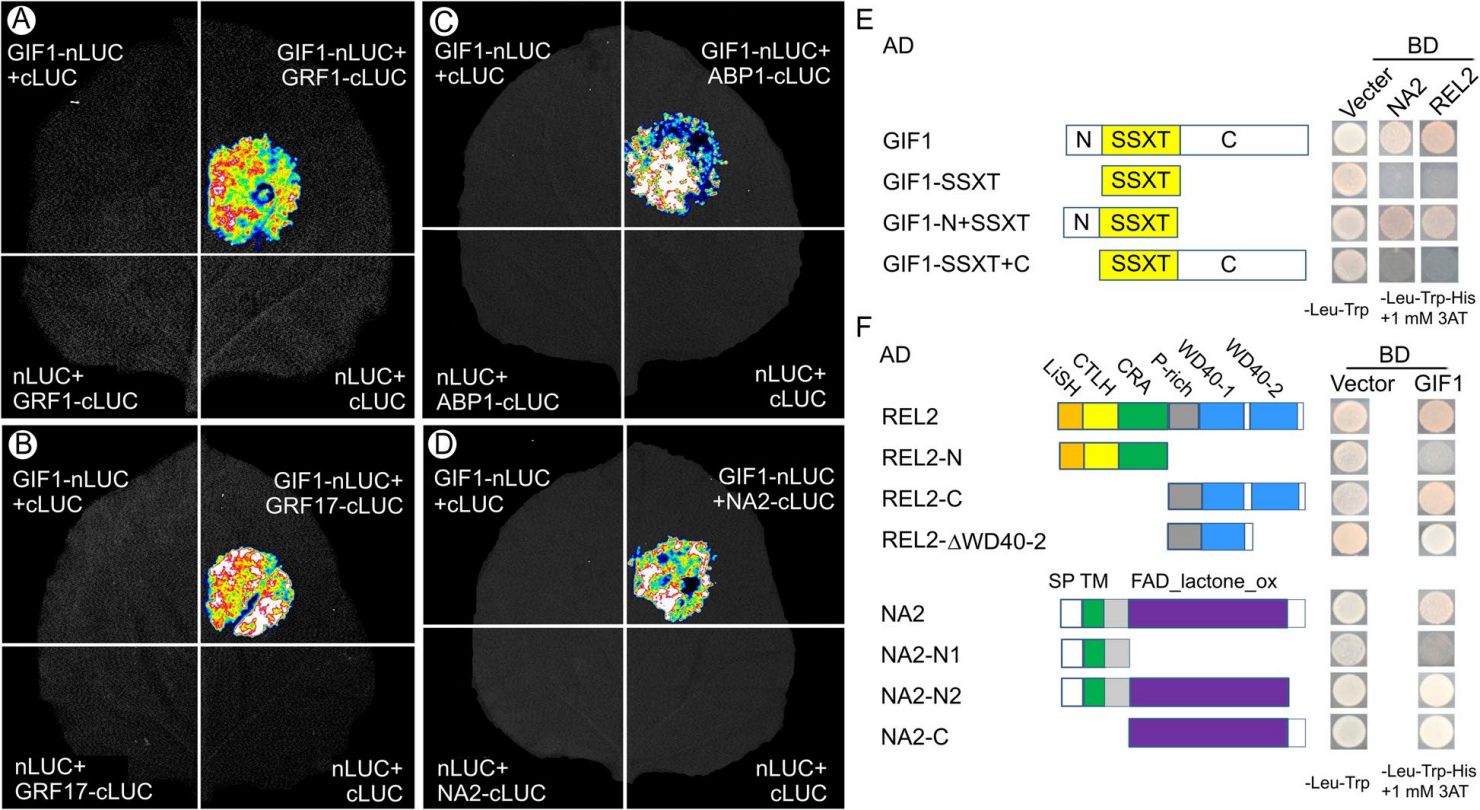
GIF1-interacting proteins and dissection of protein–protein interaction domain. **A-D** GIF1-interaction proteins revealed by firefly luciferase complementation imaging (LCI) assay. Interactions of GIF1 with GRF1 (**A**), GRF17 (**B**), ABP1 (**C**), and NA2 (**D**), respectively. **E** Both NA2 and REL2 interact with different domains of the GIF1 protein. **F** Different domains of the NA2 and REL2 interacts with the GIF1 protein. GRF: Growth-regulating factor; ABP1: Auxin-binding protein1; NA2: NANA PLANT2; REL2: RAMOSA1 ENHANCER LOCUS 2; SP: signal peptide; TM: transmembrane; Glco D: glycolate oxidase GlcD subunit

In addition to subunits of the SWI/SNF complex, Importin subunit alpha, a homologous protein of (Importin β4) that regulates Arabidopsis ovule development mediating nuclear import of GRF-interacting factors [21], was found to interact with GIF1 (Table 1). Auxin-binding protein1 (ABP1), one of putative auxin receptors, was also found to interact with GIF1 (Table 1). Notably, a brassinosteroid (BR) biosynthesis-related protein NANA PLANT2 (NA2) [39], an enzyme in jasmonic acid (JA) biosynthesis AOS1 [40, 41], and a key inflorescence factor RAMOSA1 ENHANCER LOCUS2 (REL2) [42] were found to interact in vivo with GIF1 (Table 1). Subsequently, several interaction pairs including NA2-GIF1 and ABP1-GIF1 were verified verify in vitro by firefly luciferase complementation imaging (LCI) assays in *Nicotiana benthamiana* leaves (Fig. 3A-D). These data suggest that GIF1-interacting complexes are directly and indirectly involved in diverse biological processes, including chromatin remodeling, hormone biosynthesis, and protein transport. GIF1 contains an N-terminal, a C-terminal and a SSXT domain. Furthermore, to understand the domain mediating protein interaction, we created three GIF1 constructs containing N-terminal and C-terminal truncations and performed the yeast-two-hybridization (Y2H) experiments with NA2 and REL2 proteins. We found both N-terminal and SSXT domain are required for mediating interaction with NA2 and REL2 (Fig. 3E). Similarly, a set of NA2 constructs and REL2 constructs were created as illustrated in Fig. 3F. Y2H experiments revealed that GIF1 can interact with the REL2 C-terminal domain (REL2-C), but not the N-terminal domain, the REL2 C-terminal domain lacking the WD40-2 motif can interacts with GIF1 protein as well (Fig. 3F). NA2 has a signal peptide (SP), a transmembrane region (TM), a FAD_lactone_ oxidase domain (FAD_lactone_ox), and C-terminal. Of them, the FAD_lactone_ox domain was required for mediating GIF1-NA2 interaction (Fig. 3F).

### Hormone and inflorescence development-related genes are regulated by *GIF1*

**Fig. 4:**
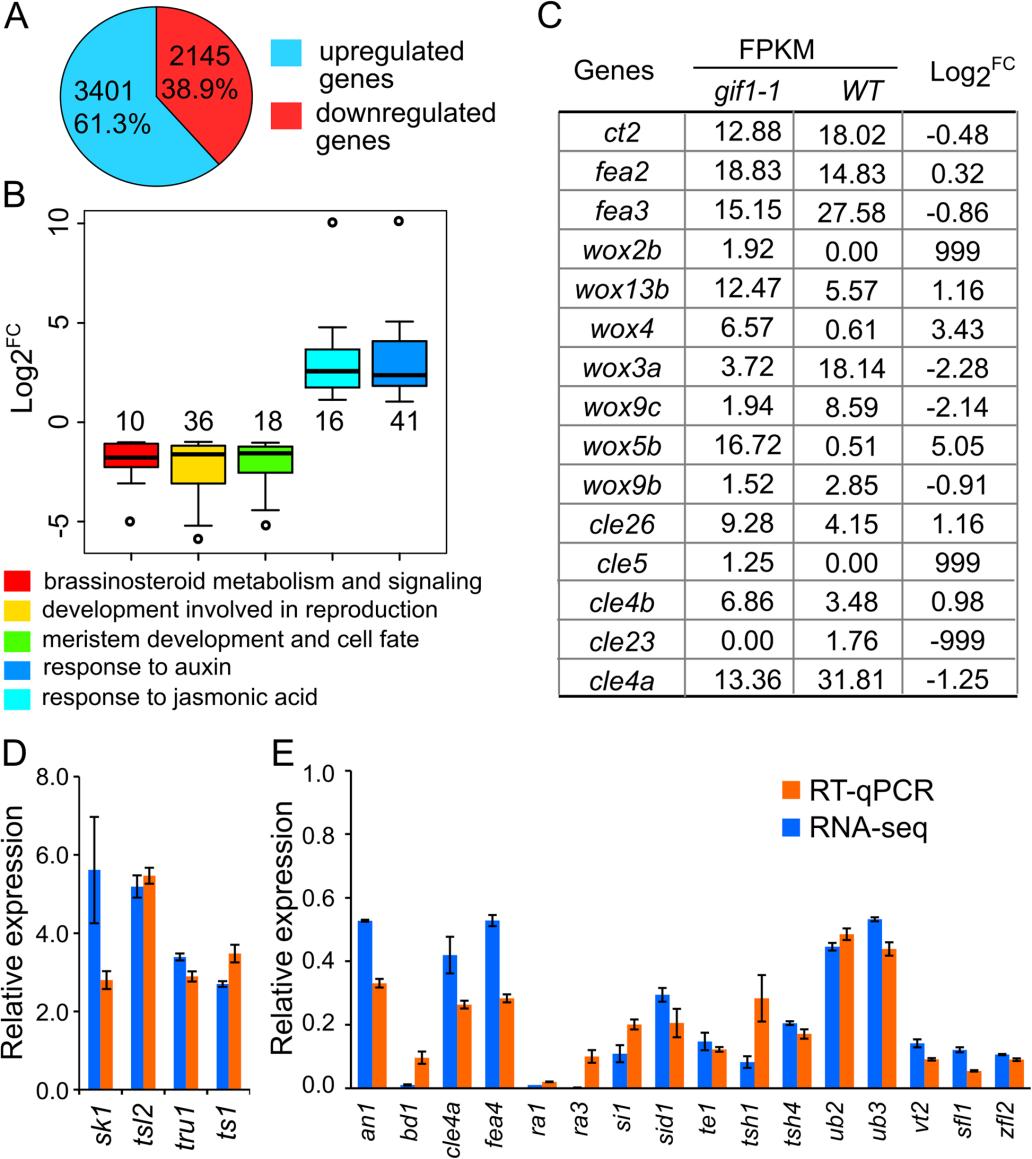
Differentially expressed genes in ears of the *gif1* mutant and wild type. **A** Number and percentage of up-regulated and down-regulated genes. **B** Representative terms of differentially expressed genes enriched in ears of the *gif1* mutant and wild type. Each box represents the median and interquartile range. Whiskers extend to maximum and minimum values. Numbers show the number of differentially expressed genes in terms. **C** Differentially expressed *WUS*-related homeobox (*wox*) genes and *CLV3*/*ENDOSPERM SURROUNDING REGION* (*ESR*)-related (*cle*) genes in ears of the *gif1* and the wild type. The 999 and -999 represent genes expressed specifically in the *gif1* and wild-type ear, respectively. **D** and **E** Relative expression levels of known inflorescence and floret-related genes. Inflorescence and floret-related genes with up-regulated expression (**D**) and down-regulated expression (**E**) in *gif1* ears. Relative expression level is detected using RNA-seq and reverse transcription quantitative PCR (RT-qPCR), respectively. RT-qPCR is performed with three biological replicates, each with three technical replicates. Fold changes in RNA transcripts are calculated by the 2^−ΔCt^ method with maize *Actin* gene (Zm00001d010159) as an internal control. All bars represent means ± s.d.; s.d.: standard deviation. FC: fold change = FPKM of a given gene detected in the *gif1*/FPKM of the gene detected in the wild-type sibling. FPKM: fragments per kilobase of transcript per million fragments mapped

To identify genes regulated by *GIF1*, we performed RNA sequencing (RNA-seq) and found that 2,145 down-regulated and 3,401 up-regulated genes were differentially expressed in 5-mm ears of the *gif1* mutant compared to that of wild-type sibling under *p* < 10^–5^ and ~ twofold expression difference (Fig. 4A, Supplemental Data Set 1). Those down-regulated genes were primarily enriched in biosynthetic process (22.5%, GO:0,009,058, *p* = 6.0e-9), biological regulation (20%, GO:0,065,007, *p* = 5.7e-9), developmental process (4.9%, GO:0,032,502, *p* = 2.7e-7) and response to stimulus (13%, GO:0,050,896, *p* = 2.8e-4) these biological processes, and nucleus (20.9%, GO:0,005,634, *p* = 5.2e-14) this cellular component term (Supplemental Data Set 2). These up-regulated genes were primarily enriched in several biological processes, including 16.0% in the single-organism metabolic process (GO:0,044,710, *p* = 3.8e-5), 9.7% in the regulation of biosynthetic process (GO:0,009,889, *p* = 2.9e-5), 9.8% in the regulation of gene expression (GO:0,010,468, *p* = 2.7e-4), 5.6% in response to chemical (GO:0,042,221, *p* = 1.4e-5), and 6.6% of up-regulated genes were enriched in the transporter activity (GO:0,005,215, *p* = 1.8e-5) molecular function (Supplemental Data Set 3).

Because of the interaction between GIF1 and NA2, we detected expression of hormone-related genes, and found that genes in BR metabolism and signaling (GO:0,016,131 and GO:0,009,742), including *nana plant1* (*Zm00001d042843*, *NA1*), *brassinosteroid-deficient dwarf1* (*Zm00001d033180*, *BRD1*), and *BR-signaling kinases* (*Zm00001d030021* and *Zm00001d047053*), were drastically down-regulated, while response to auxin (GO:0,009,733) and response to jasmonic acid (GO:0,009,753) including JA-related genes *silkless1* (Zm00001d002970, *SK1*), *tasselseed1* (Zm00001d003533, *TS1*) were up-regulated, suggesting disturbed homeostasis of hormones in the *gif1-1* ear (Fig. 4B, Supplemental Data Sets 2 and 3). Furthermore, down-regulated genes were significantly enriched in those terms for development of floret, inflorescence and meristem (Fig. 4B). In particular, genes in the CLV-WUS feedback loop were differentially expressed in ears of the *gif1-1* and wild-type plants: two *clv3*/*endosperm surrounding region*-related genes (*CLE4a* and *CLE23*) were down-regulated, while four WUS-related homeobox genes (*WOX2b*, *WOX4*, *WOX5b*, and *WOX13b*) were up-regulated in *gif1-1* ears (Fig. 4C, Supplemental Data Set 3). Importantly, a subset of well-characterized genes for inflorescence architecture including *RA1* [6, 10], *RA3* [11] *Unbranched2* (*UB2*), *UB3*, *Tassel sheath1* (*TSH1*) and *TSH4* [43, 44], and genes for floral development including *Silky1* (*SI1*) and *Silkless 1* (*SK1*) [45, 46] were down-regulated in *gif1-1* ears (Supplemental Data Set 4). Expression levels of 20 representative DEGs, which are candidates to be associated with defective phenotypes in the *gif1-1* mutant, was verified by reverse transcription-quantitative PCR (RT-qPCR) (Fig. 4D, E).

**Fig. 5:**
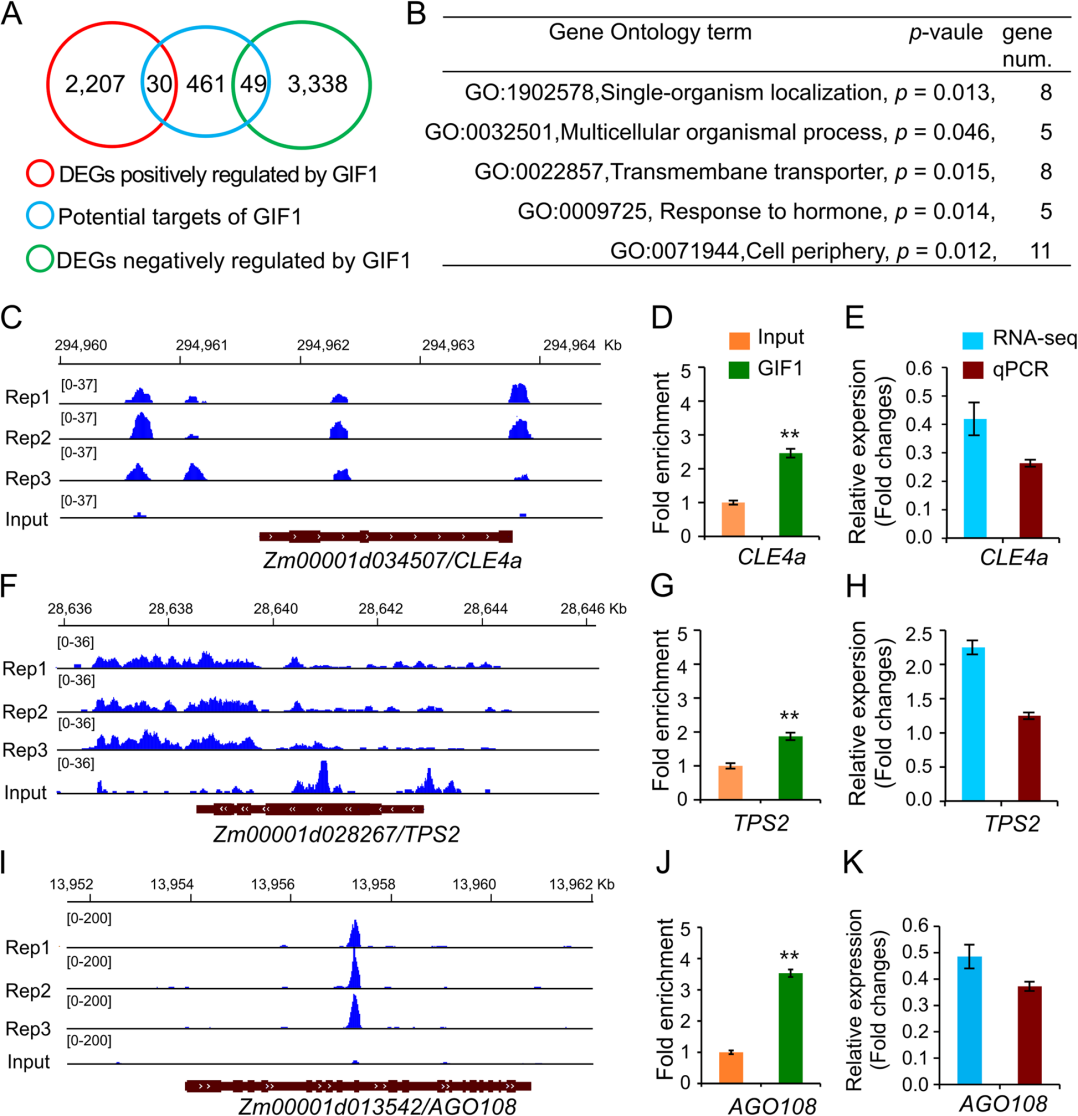
Putative targets of GIF1 detected by chromatin immunoprecipitation sequencing (ChIP-seq). **A** Genes both detected by ChIP-seq and showing differential expression detected by RNA-seq. DEG: differentially expressed gene. **B** Gene Ontology (GO) enrichment of 79 GIF1 targets. Gene num. = gene number. **C**, **F**, **I** Peak distribution of three representative targets including *CLE4a* C, *TPS2* F and *AGO108* (**I**). **D**, **G**, **J** Fold enrichment of three representative targets detected by ChIP-qPCR. Gene specific primers were used to quantify DNA targets including *CLE4a* (D), *TPS2* (**G**) and *AGO108* (**J**) immunoprecipitated by anti-GFP antibody relative to input DNA, respectively. The columns are the mean value of fold enrichment detected in three separate experiments, each with three technical replicates. Error bars show the standard deviation. The statistical significance was estimated using a Student’s t-test. ** *P* < 0.01. **E**, **H**, **K** Relative expression levels of *CLE4a* (**E**), *TPS2* (**H**) and *AGO108* (**K**) in ears of the *gif1* and the wild type detected by RNA-seq and qPCR. qPCR is performed with three biological replicates, each with three technical replicates. Error bars show the standard deviation. CLE4a: CLAVATA3 (CLV3)/ENDOSPERM SURROUNDING REGION (ESR) 4a. TPS2: Trehalose-6-phosphate synthase 2. AGO108: Argonaute108

To determine the occupancy of GIF1, we performed ChIP-seq to detect GIF1-bound DNA regions in immature ears (~ 5 mm) of transgenic line OE2 overexpressing *GIF1-GFP* using anti-GFP antibody. A total of 10,460 high-confidence peaks were identified by comparing significantly GIF1-enriched peaks with the input control (*p* < 10^–5^), of which, 1,308 peaks were shared in at least two replicates, respectively (Supplemental Fig. 2). GIF1 bound in various genomic contexts, with a high proportion (45.0%) of binding within intergenic regions which agree with the interaction between GIF1 and SWI/SNF subunits, and 12.9% and 11.9% binding within 1.0 kb downstream of the terminal site and within exons, respectively (Supplemental Fig. 2). Within 10 kb of high-confidence peaks, we identified 540 genes as putative targets of GIF1 in at least two replicates (Supplemental Fig. 2). Furthermore, 79 DEGs including 47 down-regulated (59.5%) and 32 up-regulated (40.5%) genes in *gif1* ears were bound by GIF1 (Fig. 5A, Supplemental Table 2), suggesting that these genes are direct targets of GIF1, which acts as a repressor and an activator of gene expression in developing ears. The function of *GIF1* in repression of gene expression can be partially explained by its interactors (Table 1), such as subunits of SWI/SNF chromatin remodeling complex, and RAMOSA1 ENHANCER LOCUS2 (REL2) which is a transcriptional co-repressor functioning in vegetative and reproductive architecture [42]. Notably, these targets of GIF1 were significantly enriched in 5 GO terms including cell periphery (GO: 0,071,944) and response to hormone (GO: 0,009,725) (Fig. 5B). In addition, several meristem identify, determinacy and maintenance-related genes were also bound by GIF1 (Supplemental Table 2). For example, GIF1 bound to the promoter and 3′-untranslated region (UTR) of *CLE4a*, the promoter region of *TPS2*, gene body of *AGO108*. As expression of *CLE4a* and *AGO108* was significantly down-regulated in *gif1* ears (Fig. 5C-E,I-K), the data suggest that *CLE4a* and *AGO108* are two positively regulated targets of GIF1. The expression of *TPS2* was significantly up-regulated in *gif1* ears (Fig. 5F-H), indicating that *TPS2* is a negatively regulated target of GIF1. Moreover, *PIN8*, *RA2*, *OFP21* (Supplemental Fig. 3) and several TF-encoding genes (Supplemental Table 2) were also strong candidates for key targets of GIF1.

## Discussion

**Fig. 6:**
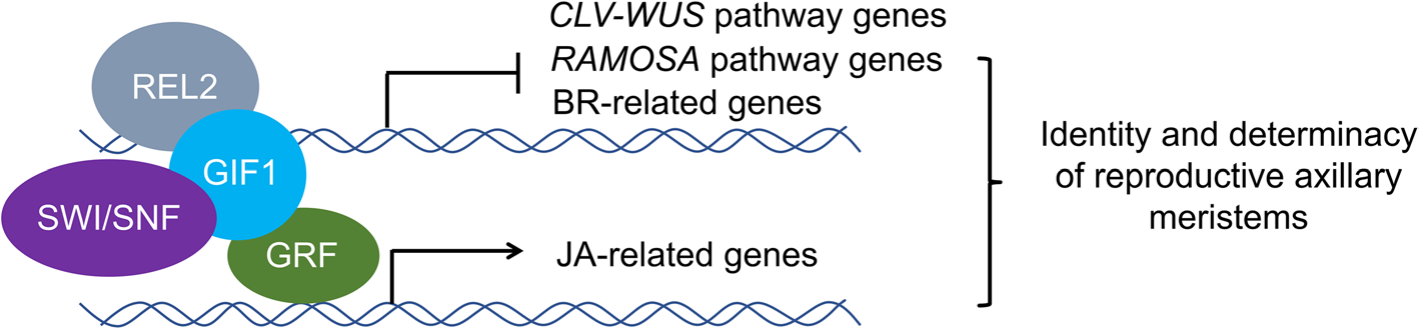
A possible working mechanism for GIF1 regulating meristem identity and determinacy in the ear inflorescence of maize

To explore the role of *GIF1* regulation in ear meristems, we created *GIF1-*overexpressing lines to complement *gif1* and identified GIF1-interacting proteins and GIF1 target genes. We found that a set of GRFs and some subunits of the SWI/SNF complex interact with GIF1 in vivo, the finding is consistent with those results from early investigations on GIF1-interacting protein in vegetative and reproductive development of Arabidopsis [16, 18], rice [26, 27], and maize [35]. SWI/SNF are high molecular weight complexes that could change interactions between histone octamers and the DNA [38]. GIF1 working together with GRFs recruits SWI/SNF chromatin-remodeling ATPases to influence DNA accessibility and might expose the cis-element of target genes to GRFs. GRFs could stimulate or inhibit the transcription of target genes (Fig. 6). In addition to GRFs and SWI/SNF factors, we found that GIF1 interacts with proteins that involve in cell division, molecular signaling, etc. Thus, we suggest that GIF1 is involved in a wide spectrum of biological processes by selectively interacting with diverse proteins during ear development.

GIF1 regulates the fate of ear axillary meristems and floral organ primordia in ear inflorescence of maize. We found that GIF1 could regulate identity and determinacy of reproductive axillary meristems by hormone biosynthesis process (Fig. 6). We found that BR biosynthesis enzyme NA2 interacts with GIF1*,* and 10 genes related with BR metabolism and signaling pathway including *BRD1* and *NA1* were significantly down-regulated in the *gif1* mutant. BR is found to affect plant height, branching, and sexual organ (stamen and pistil) development in maize [39, 47, 48]. The severely reduced plant height and ear with anthers in *na2* mutants was similar to dwarf plant and highly frequent branches in *gif1* mutants. We also found that JA-related genes *SK1*, *TS1* and *TS2* were up-regulated in *gif1* ears. Both *TS1* and *TS2* are required for JA-mediated elimination of pistils in the staminate [49, 50]. Conversely, *SK1* could protect pistils in the ear florets from JA-mediated elimination [46]. The up-regulated JA-related genes might participate in the identity of SMs and FMs in the *gif1* mutant. Therefore, we propose that BR-related and JA-related pathway for floral organ development and meristem identity are regulated by *GIF1* (Fig. 6).

GIF1 also regulates BM determinacy by targeting *RAMOSA* and *CLV-WUS* pathway. The *gif1* mutant produced ear with long branches similar to that of *ramosa* mutants. The well characterized *RAMOSA* genes, *RA1*, *RA2*, *RA3* and *REL2* are involved in AM formation and BM determinacy [6, 10, 11, 51]. We found that GIF1 is directly interacted with REL2, an enhancer of *RA1*. The GIF1-REL2 complex might regulate the BM determinacy by *RA1*. *GIF1* is a positive regulator of *RA2* but a negative regulator of *TPS2*. *TPS2* encodes a trehalose-6-phosphate (T6P) synthase. RA3 controls long branches in the ear and the tassel by catalyzing dephosphorylation of T6P [6]. Thus, we infer that GIF1 regulates AM determinacy with RAMOSA pathway genes. The *CLAVATA* genes encode CLV ligands and CLV receptors. CLV3, a small peptide ligand secreted from cells of the central zone, is perceived by CLV1 and CLV2 to repress *WUS* transcription to regulate the meristem size [52]. Two maize CLV3 orthologs secreted peptides, ZmCLE7 and ZmFCP1, interact with CLV2 ortholog FEA2 to transmit signals and regulate inflorescence meristem size [53]. We found that GIF1 binds to the promoter of *CLE4a* and down-regulated the expression of *CLE4a* in the *gif1* mutant, suggesting that *GIF1* is a positive regulator of *CLE4a* and the IM activity of *gif1* ears might result from transcriptional repression of *CLE4a*.

## Conclusions

The transcription coactivator Growth-regulating factor (GRF)-interacting factor 1 (GIF1) interacts with given GRFs dependent upon the developmental context, and also recruit additional protein factors, for example SWI/SNF chromatin remodeling complexes, Ramosa1 Enhancer Locus 2 (REL2), to establish a multi-factor transcription complex. The transcription complex specifically bind to its target genes to repress Brassinolide (BR) biosynthesis and metabolism genes (*BRD1* and *NA1*); meristem maintenance gene *CLE4a* and axillary meristem determinacy gene *TPS*, but activate Jasmonic acid (JA) biosynthesis genes (*SK1*, *TS1* and *TS2*). Consequently, the fine transcription control of these target genes determines the identity and determinacy of reproductive axillary meristems in the ear inflorescence (Fig. 6).

## Supplementary Information

**Additional file 1:** **Supplemental Figure 1.** Identification of total proteins and immunoprecipitated proteins by sodium dodecyl sulfate-polyacrylamide gel electrophoresis (SDS-PAGE) and immunoblotting. **Supplemental Figure 2.** Summary of chromatin immunoprecipitation sequencing (ChIP-seq). **Supplemental Figure 3.** Targets of GIF1 detected by chromatin immunoprecipitation sequencing (ChIP-seq). **Supplemental Figure 4.** Schematic diagram of the gif1 over-expression construct. The construct components include the T-DNA right border, RB; and left border, LB; CaMV35S promoter, CaMV35S; terminator of nopaline synthase gene, tnos; enhanced green fluorescent protein gene, eGFP; the phosphinothricin acetyltransferase cassette, bar. **Supplemental Table 1.** Conservation of identified proteins in Arabidopsis, maize leaf and maize ear. **Supplemental. Table 2.** Putative GIF1-bound targets identified by ChIP-seq and RNA-seq. **Supplemental Table 3.** Primer sequences used in this study.

**Additional file 2:** **Supplemental Data Set 1.** A list of differentially expressed genes and their expression levels. Gene expression is calculated using the FPKM (Fragments per kilobase transcriptome per million mapped) method. Fold change: average FPKM of given gene in gif1 mutant / that in wild type. The differentially expression genes (DEGs) are identified using *p*<10-5 and q <10-4. Rep: biological replicate.

**Additional file 3:** **Supplemental Data Set 2.** Gene ontology enrichment of down-regulated differentially expressed genes. GO_acc: GO accession; term_type includes molecular function (F), cellular component (C), biological process (P); queryitem: item number mapping the GO in the query list; querytotal: total number of query list; bgitem: item number mapping the GO in the background; bgtotal: total number of background; FDR: Yekutieli (FDR under dependency).

**Additional file 4:** **Supplemental Data Set 3.** Gene ontology enrichment of up-regulated differentially expressed genes. GO_acc: GO accession; term_type includes molecular function (F), cellular component (C), biological process (P); queryitem: item number mapping the GO in the query list; querytotal: total number of query list; bgitem: item number mapping the GO in the background; bgtotal: total number of background; FDR: Yekutieli (FDR under dependency).

**Additional file 5:** **Supplemental Data Set 4.** Representative GO terms of differentially expressed genes in the ~5 mm ears of the gif1-1 and the wild type.

## Data Availability

Sequence data from this article can be found in the NCBI SRA dataset under the following accession numbers: PRJNA790036.

## Abbreviations

BM: Branch meristem
BR: Brassinolide
ChIP: Chromatin immunoprecipitation
DEG: Differentially expressed gene
FEA: Fasciated ear
FM: Floral meristem
GFP: Green fluorescent protein
GIF: Growth-regulating factor-interacting factor
GO: Gene ontology
GRF: Growth-regulating factor
HRP: Horseradish peroxidase
IM: Inflorescence meristem
IP-MS: Immunoprecipitation-mass spectrometry
JA: Jasmonic acid
LCI: Firefly luciferase complementation imaging
LOB: Lateral organ boundary
NT: Non-transgenic sibling
OE: Overexpression line
ORF: Open reading frame
SDS-PAGE: Sodium dodecyl sulfate–polyacrylamide gel electrophoresis
SM: Spikelet meristem
SPM: Spikelet pair meristem
T6P: Trehalose 6-phosphate
TF: Transcription factor
UTR: 3′-Untranslated region
Y2H: Yeast-two-hybridization
