# Supplementary material for: GIF1 controls ear inflorescence architecture and floral development by regulating key genes in hormone biosynthesis and meristem determinacy in maize
Source: BMC Plant Biol. 2022 Mar 18;22:127. doi: 10.1186/s12870-022-03517-9 (PMC8932133; doi:10.1186/s12870-022-03517-9)
Supplement: Supplementary file 1 — Additional file 1: Supplemental Figure 1. Identification of total proteins and immunoprecipitated proteins by sodium dodecyl sulfate-polyacrylamide gel electrophoresis (SDS-PAGE) and immunoblotting. Supplemental Figure 2. Summary of chromatin immunoprecipitation sequencing (ChIP-seq). Supplemental Figure 3. Targets of GIF1 detected by chromatin immunoprecipitation sequencing (ChIP-seq). Supplemental Figure 4. Schematic diagram of the gif1 over-expression construct. The construct components include the T-DNA right border, RB; and left border, LB; CaMV35S promoter, CaMV35S; terminator of nopaline synthase gene, tnos; enhanced green fluorescent protein gene, eGFP; the phosphinothricin acetyltransferase cassette, bar. Supplemental Table 1. Conservation of identified proteins in Arabidopsis, maize leaf and maize ear. Supplemental. Table 2. Putative GIF1-bound targets identified by ChIP-seq and RNA-seq. Supplemental Table 3. Primer sequences used in this study. [file 12870_2022_3517_MOESM1_ESM.zip › gel and blot images.docx]

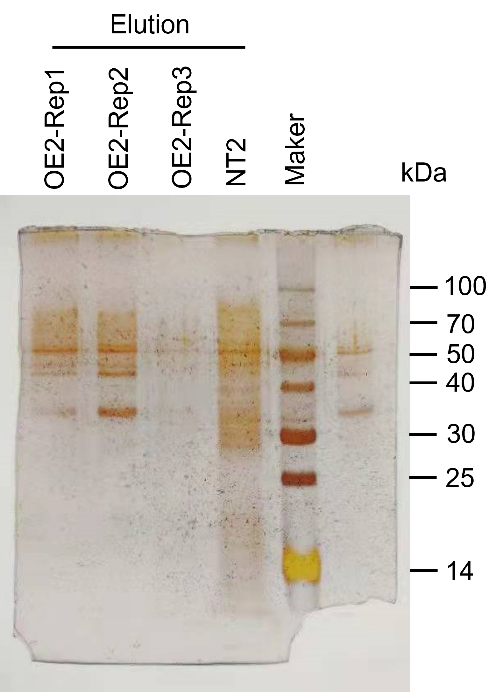

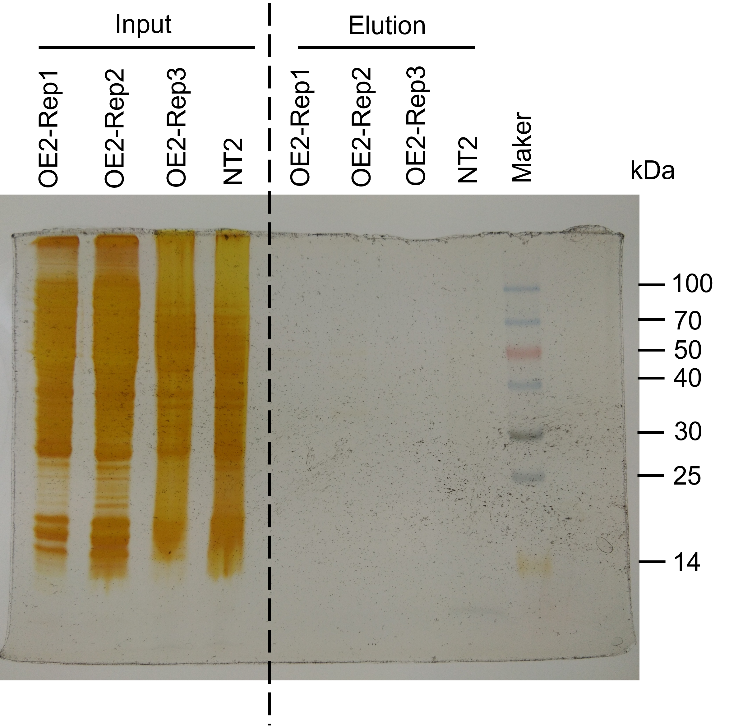


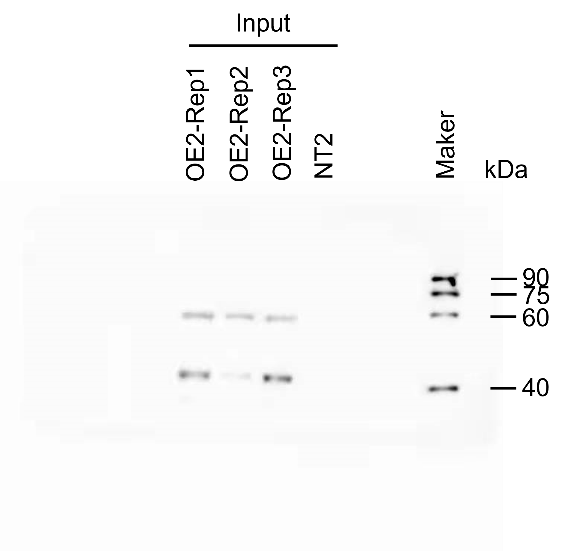


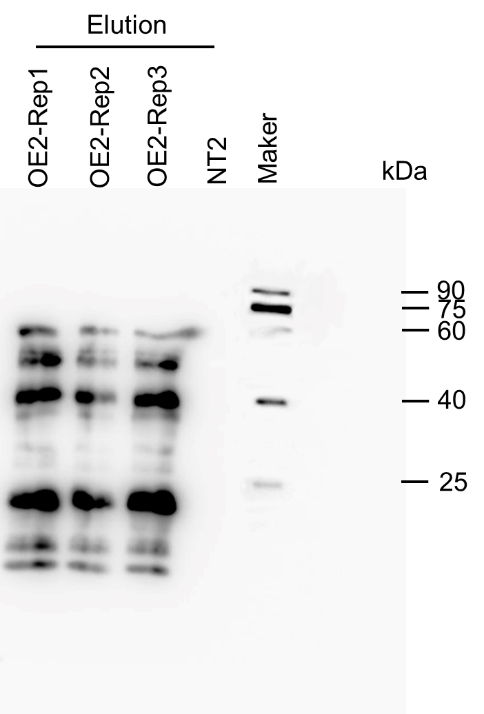


**Supplemental Figure 1**. Identification of total proteins and immunoprecipitated proteins by sodium dodecyl sulfate-polyacrylamide gel electrophoresis (SDS-PAGE) and immunoblotting.

Total proteins extracted from 5 mm ears of transgenic line OE2 overexpressing GIF1-GFP and separated by SDS-PAGE. Proteins are visualized using silver staining. GIF1-binding proteins immunoprecipitated by mouse monoclonal anti-GFP (Green Fluorescent Protein) antibody. Proteins from corresponding non-transgenic sibling line (NT2) are used as a negative control.

Note: We cut the “Elution part” of the gel and stained the gel again with silver to get
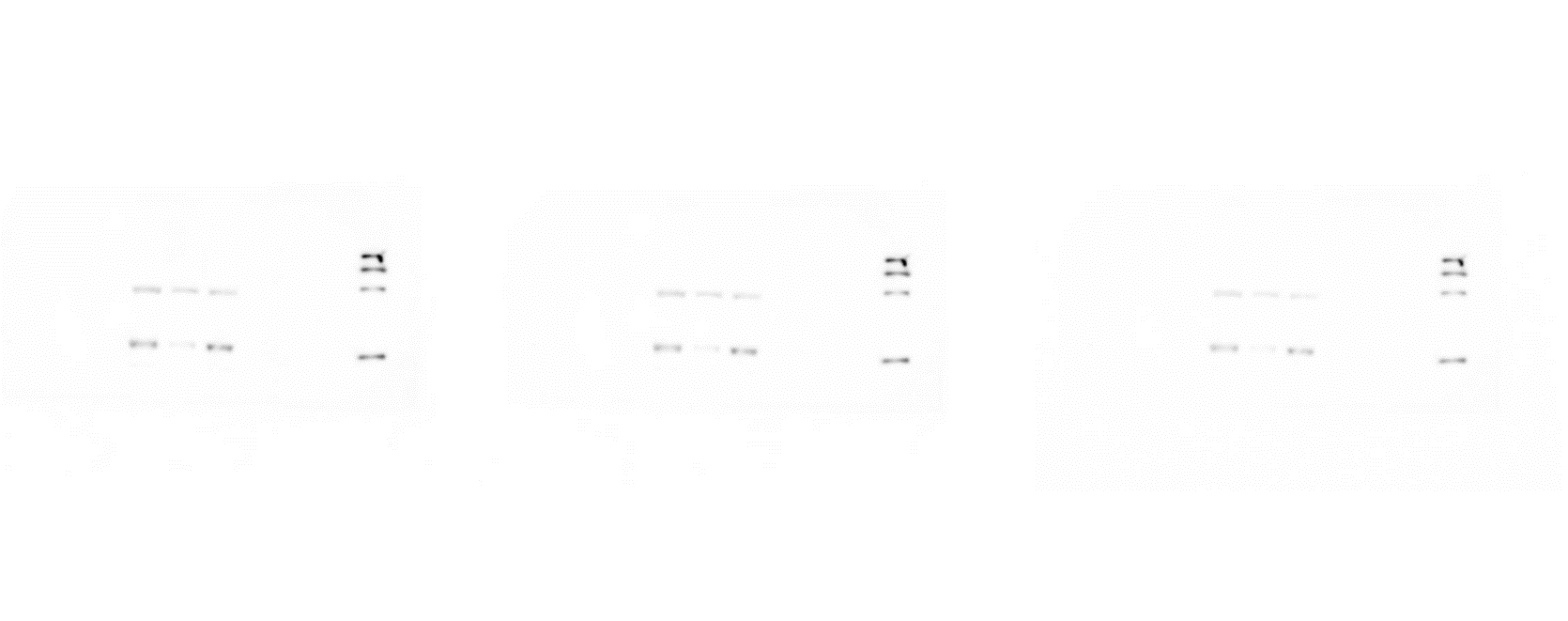
the weak band of elution protein.

Multiple exposure images for Supplemental Figure 1-anti-GFP-Input.


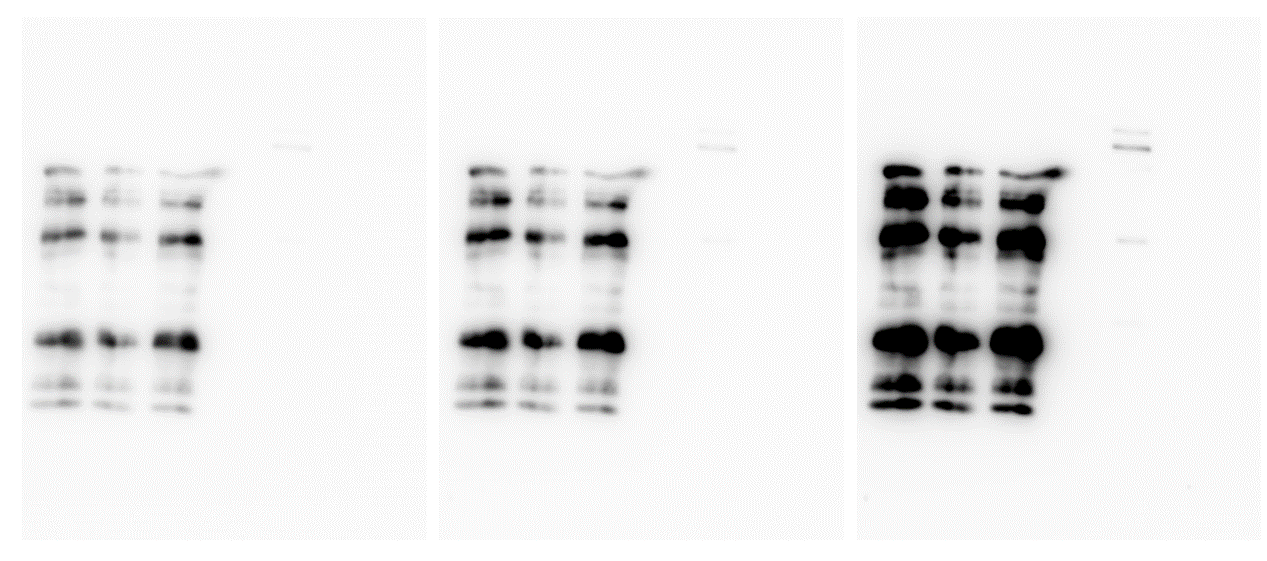


Multiple exposure images for Supplemental Figure 1-anti-GFP-Elution.
