# Supplementary material for: GIF1 controls ear inflorescence architecture and floral development by regulating key genes in hormone biosynthesis and meristem determinacy in maize
Source: BMC Plant Biol. 2022 Mar 18;22:127. doi: 10.1186/s12870-022-03517-9 (PMC8932133; doi:10.1186/s12870-022-03517-9)
Supplement: Supplementary file 1 — Additional file 1: Supplemental Figure 1. Identification of total proteins and immunoprecipitated proteins by sodium dodecyl sulfate-polyacrylamide gel electrophoresis (SDS-PAGE) and immunoblotting. Supplemental Figure 2. Summary of chromatin immunoprecipitation sequencing (ChIP-seq). Supplemental Figure 3. Targets of GIF1 detected by chromatin immunoprecipitation sequencing (ChIP-seq). Supplemental Figure 4. Schematic diagram of the gif1 over-expression construct. The construct components include the T-DNA right border, RB; and left border, LB; CaMV35S promoter, CaMV35S; terminator of nopaline synthase gene, tnos; enhanced green fluorescent protein gene, eGFP; the phosphinothricin acetyltransferase cassette, bar. Supplemental Table 1. Conservation of identified proteins in Arabidopsis, maize leaf and maize ear. Supplemental. Table 2. Putative GIF1-bound targets identified by ChIP-seq and RNA-seq. Supplemental Table 3. Primer sequences used in this study. [file 12870_2022_3517_MOESM1_ESM.zip › Supplementary file.docx]

Supplemental information

GIF1 controls ear inflorescence architecture and floral development by regulating key genes in hormone biosynthesis and meristem determinacy in maize

Manfei Li^1,2,#^ ,Yuanyuan Zheng^2,#^, Di Cui^2^, Yanfang Du^2^, Dan Zhang^3^, Wei Sun^2^, Hewei Du^1,*^, Zuxin Zhang^2,*^

^1^ College of Life Science, Yangtze University, Jingzhou 434025, P.R. China

^2^ National Key Laboratory of Crop Genetic Improvement, Hubei Hongshan Laboratory, Huazhong Agricultural University, Wuhan 430070, P.R. China

^3^ College of Agronomy, Tarim University, Alar, Xinjiang 843300, P.R. China

^#^ These authors contributed equally to this work.

^*^ Author for correspondence:

Zuxin Zhang

Tel: +86 027 87282689

Email: [zuxinzhang@mail.hzau.edu.cn](mailto:zuxinzhang@mail.hzau.edu.cn)

Hewei Du

Email: [200457@yangtzeu.edu.cn](mailto:200457@yangtzeu.edu.cn)


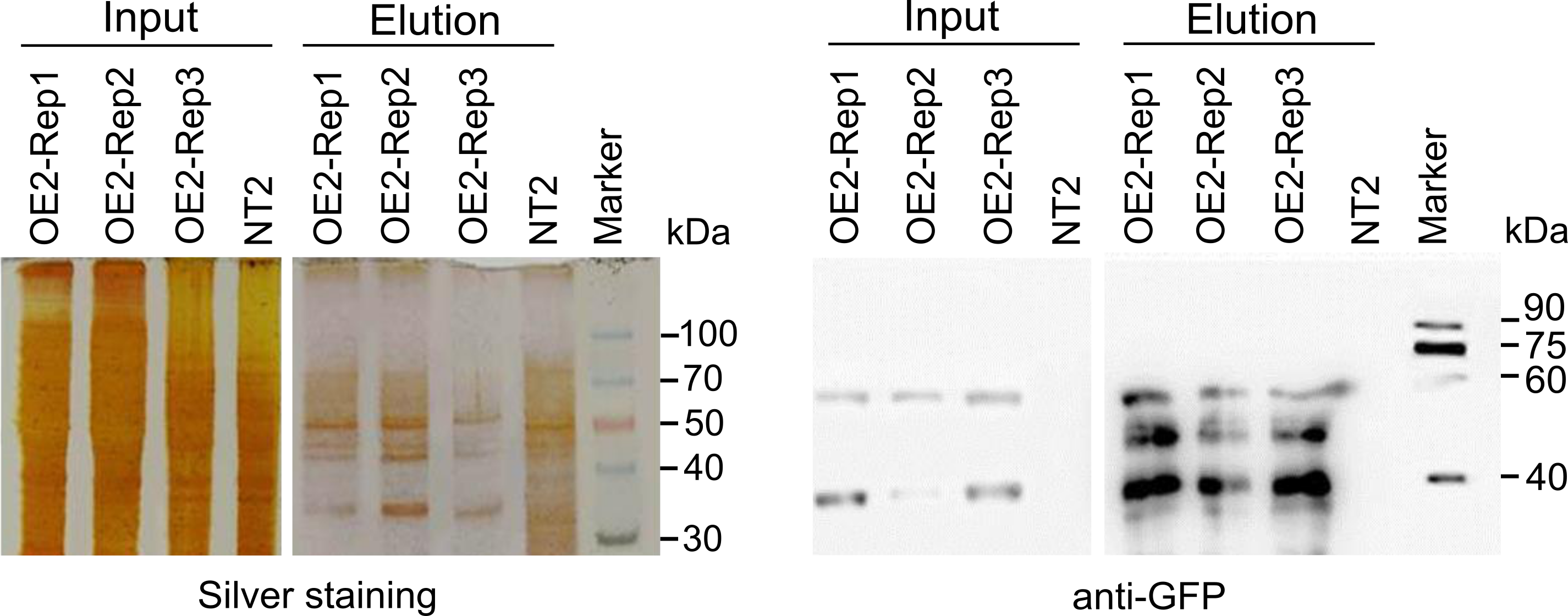


**Supplemental Figure 1**. Identification of total proteins and immunoprecipitated proteins by sodium dodecyl sulfate-polyacrylamide gel electrophoresis (SDS-PAGE) and immunoblotting.

Total proteins extracted from 5 mm ears of transgenic line OE2 overexpressing GIF1-GFP and separated by SDS-PAGE. Proteins are visualized using silver staining. GIF1-binding proteins immunoprecipitated by mouse monoclonal anti-GFP (Green Fluorescent Protein) antibody. Proteins from corresponding non-transgenic sibling line (NT2) are used as a negative control.


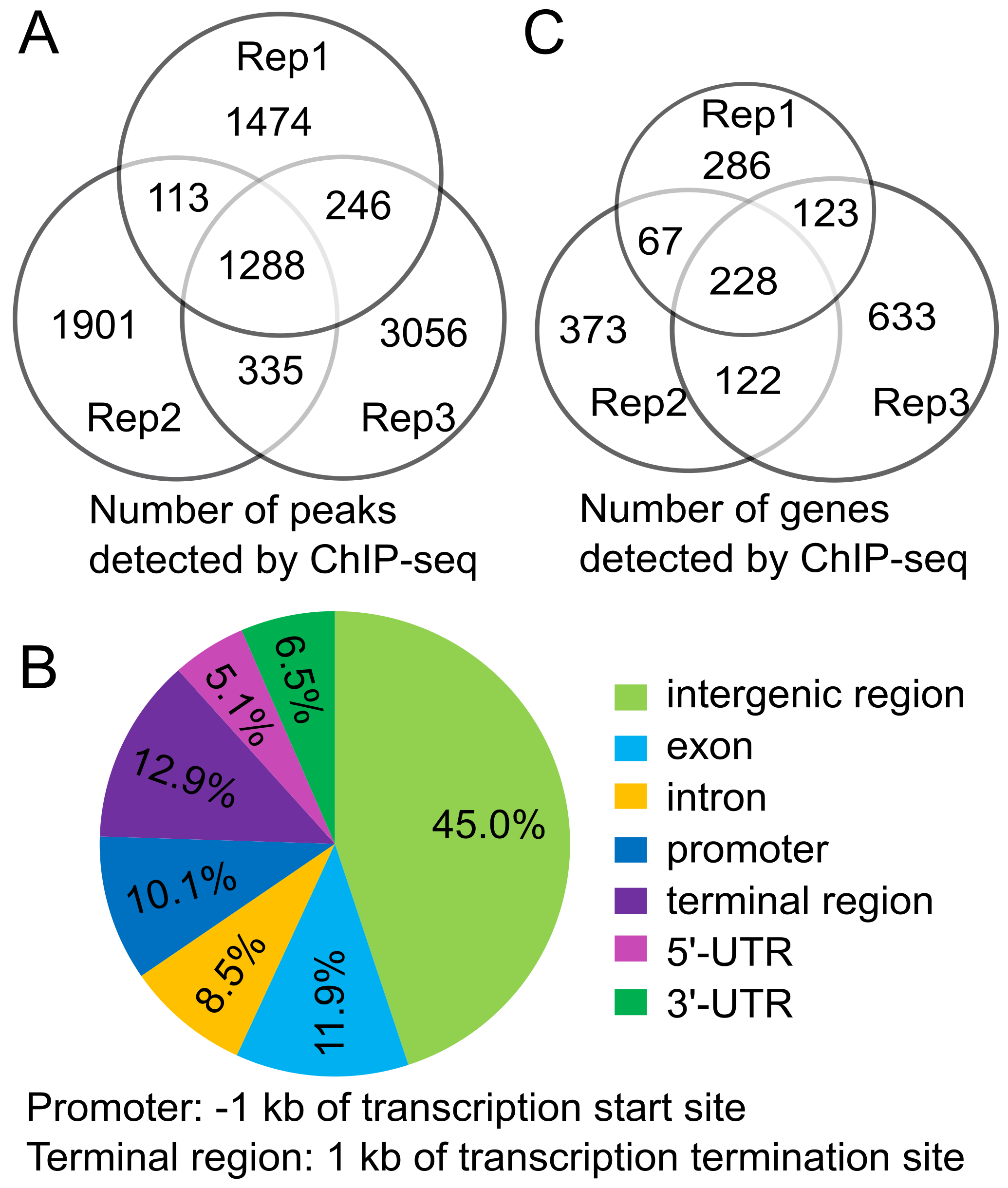


**Supplemental Figure 2**. Summary of chromatin immunoprecipitation sequencing (ChIP-seq).

A) Peak number detected in three biological replicates.

B) Distribution of peaks detected by ChIP-seq in the genomic contexts.

C) gene number (right panel) detected in three biological replicates.


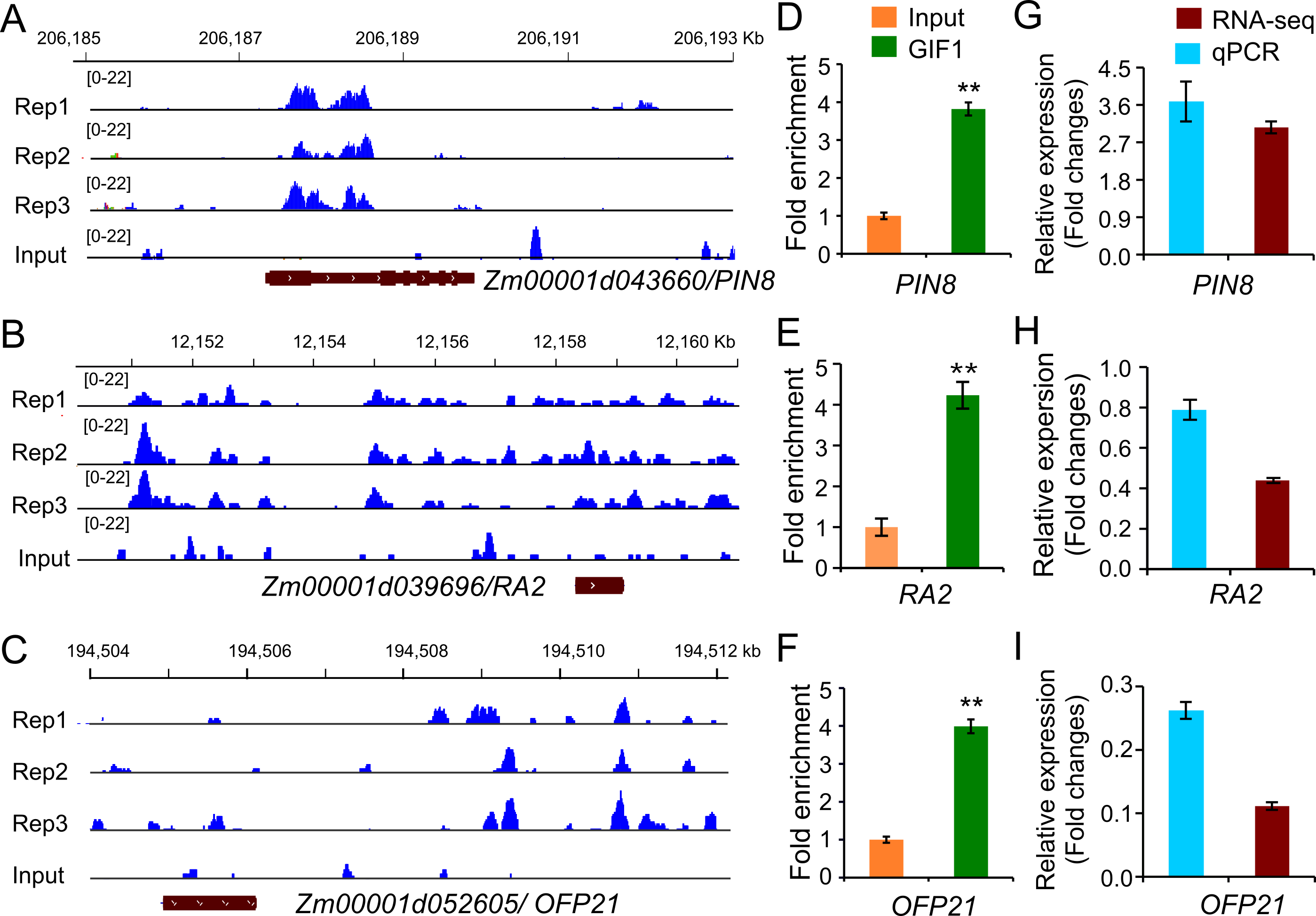


**Supplemental Figure 3.** Targets of GIF1 detected by chromatin immunoprecipitation sequencing (ChIP-seq).

(A-C) Peak distribution of three representative targets: *PIN8 (A)*, *RA2* (*RAMOSA2*) (B) and *OFP21* (*OVATE-transcription factor 21*) (C).

(D-F) Fold enrichment of three representative targets: *PIN8 (D)*, *RA2* (E) and *OFP21* (F) detected by ChIP-qPCR.

(G-I) Relative expression of *PIN8 (G)*, *RA2* (H) and *OFP21* (I) in ears of the *gif1-1* and wild-type sibling detected by RNA-seq and reverse transcription-quantitative PCR (RT-qPCR). qPCR is performed with three biological replicates, each with three technical replicates. Error bars show the standard deviation.


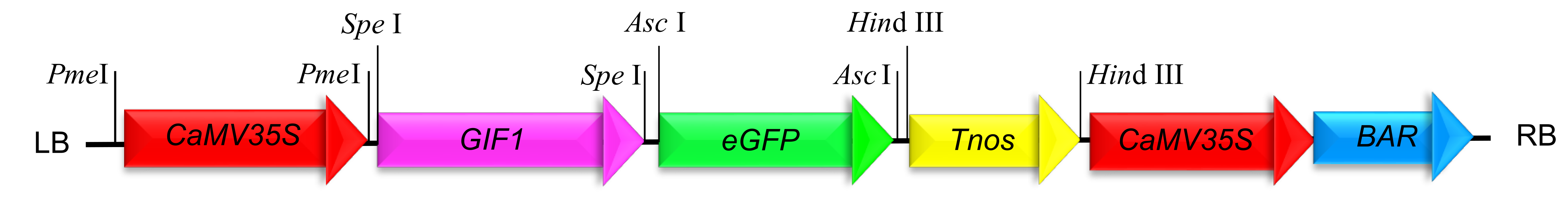


**Supplemental Figure 4.** Schematic diagram of the *gif1* over-expression construct. The construct components include the T-DNA right border, RB; and left border, LB; *CaMV35S* promoter, *CaMV35S;* terminator of nopaline synthase gene, *tnos*; enhanced green fluorescent protein gene, *eGFP*; the phosphinothricin acetyltransferase cassette, *bar*.

**Supplemental Table 1**. Conservation of identified proteins in Arabidopsis, maize leaf and maize ear

| Function or domain | Arabidopsis | Maize leaf  -TAP | Maize ear  -TAP | Maize ear  -IP |
| --- | --- | --- | --- | --- |
| ATPase  Helicase | RM  SYD | RM  SYD | BRM  SYD | RRM/RBD/RNP  RH2, MCM |
| Actin-like | ARP4, ARP7 | ARP4, ARP7 | SWI3D | ARP4, ARP7 |
| SWI/SNF subunit | SWI3C, SWI3D  SWI3B, SWP73 | SWI3C, SWI3D  SWP73 | SNF12 | SWI3D  SNF12 |
| AT-rich interactive domain | LFR | LFR | – | – |
| Agenet domain | – | G2484-1 | G2484-1 | G2484-1 |
| LEUNG HOMOLOG | – | LUH | LUH | – |
| Dentin sialophosphoprotein  -related protein | – | Dentin sialophospho | Dentin sialophospho | – |
| GRF | GRF1, GRF2, GRF3,GRF4, GRF5, GRF6,  GRF7, GRF10, GRF14,GRF15, GRF17, GRF18 | GRF1, GRF3, GRF4,GRF5, GRF6, GRF7, GRF10, GRF17 | GRF1, GRF2, GRF3,GRF5, GRF6, GRF7,  GRF10, GRF11, GRF12, GRF14, GRF15, GRF17,  GRF18 | GRF1, GRF4, GRF7,GRF10, GRF11, GRF12, GRF15, GRF17, GRF8 |

Data were compared with data obtained in Arabidopsis (Vercruyssen et al., 2014) and maize (Nelissen et al., 2015). TAP: tandem affinity purification

**Supplemental Table 2**. Putative GIF1-bound targets identified by ChIP-seq and RNA-seq

| ID | Gene accession | Anotation | # of replicates detected |
| --- | --- | --- | --- |
| 1 | Zm00001d001772 | Protein YLS9 | 2 |
| 2 | Zm00001d003774 | oxidoreductase superfamily protein | 2 |
| 3 | Zm00001d004744 | Transcription repressor MYB6 | 2 |
| 4 | Zm00001d006417 | RING/U-box superfamily protein | 3 |
| 5 | Zm00001d006697 | Putative polyol transporter 1 | 3 |
| 6 | Zm00001d008205 | phytochrome interacting factor3 | 3 |
| 7 | Zm00001d009060 | beta-D-xylosidase 5 | 2 |
| 8 | Zm00001d009762 | DCDdomain protein | 2 |
| 9 | Zm00001d009932 | Plasma membrane associated protein | 3 |
| 10 | Zm00001d011174 | senescence enhanced2a | 2 |
| 11 | Zm00001d011673 | farnesyl diphosphate synthase2 | 2 |
| 12 | Zm00001d011874 | Plant calmodulin-binding protein-related | 2 |
| 13 | Zm00001d012402 | avr9/Cf-9 rapidly elicited protein | 2 |
| 14 | Zm00001d013271 | RNA binding protein | 2 |
| 15 | Zm00001d013498 | Protein yippee-like | 3 |
| 16 | Zm00001d013542 | argonaute108 | 3 |
| 17 | Zm00001d013825 | expressed protein | 3 |
| 18 | Zm00001d014368 | Chaperone DnaJ-domain superfamily protein | 3 |
| 19 | Zm00001d016681 | PAN domain-containing protein | 2 |
| 20 | Zm00001d016682 | RING-H2 finger protein ATL7 | 2 |
| 21 | Zm00001d017547 | Dehydrin COR410 | 2 |
| 22 | Zm00001d017907 | Protein TIC 20-v chloroplastic | 3 |
| 23 | Zm00001d018085 | hypothetical protein | 2 |
| 24 | Zm00001d021647 | ABC transporter G family member 34 | 2 |
| 25 | Zm00001d021903 | beta-glucosidase 4 | 2 |
| 26 | Zm00001d022141 | DNA-directed RNA polymerase subunit | 3 |
| 27 | Zm00001d022395 | Rhythmically expressed protein | 3 |
| 28 | Zm00001d023421 | hypothetical protein | 2 |
| 29 | Zm00001d024830 | Protease Do-like 2 chloroplastic | 3 |
| 30 | Zm00001d025572 | Sugar transport protein 5 | 3 |
| 31 | Zm00001d026055 | glycosyltransferase-related family protein | 2 |
| 32 | Zm00001d026182 | Ethylene-responsive transcription factor | 2 |
| 33 | Zm00001d026528 | Oligouridylate-binding protein 1C | 2 |
| 34 | Zm00001d028267 | trehalose-6-phosphate synthase2 | 3 |
| 35 | Zm00001d031653 | Glycerophosphodiester phosphodiesterase | 2 |
| 36 | Zm00001d032279 | ABC2 homolog 15 | 2 |
| 37 | Zm00001d032501 | Endonuclease 1 | 3 |
| 38 | Zm00001d032926 | chlorophyllase2 | 3 |
| 39 | Zm00001d033065 | expressed protein | 3 |
| 40 | Zm00001d033468 | glyoxalase I family protein | 3 |
| 41 | Zm00001d033878 | ACT domain-containing protein ACR1 | 2 |
| 42 | Zm00001d034507 | CLE4a | 3 |
| 43 | Zm00001d034655 | Probable protein phosphatase 2C | 2 |
| 44 | Zm00001d035651 | DNA binding with one finger3 | 3 |
| 45 | Zm00001d036690 | OSJNBa0041A02.21 protein | 3 |
| 46 | Zm00001d038146 | 2OG-Fe oxygenase family protein | 3 |
| 47 | Zm00001d038180 | Protein NRT1/ PTR FAMILY 3.1 | 2 |
| 48 | Zm00001d039293 | tonoplast intrinsic protein4 | 2 |
| 49 | Zm00001d039492 | myb-like transcription factor family protein | 2 |
| 50 | Zm00001d039606 | Serine and sphingolipid biosynthesis protein | 3 |
| 51 | Zm00001d039634 | dwarf plant1 | 2 |
| 52 | Zm00001d039685 | Galactinol-sucrose galactosyltransferase 5 | 2 |
| 53 | Zm00001d039867 | Factor of DNA methylation 4 | 2 |
| 54 | Zm00001d039987 | MYB family transcription factor | 2 |
| 55 | Zm00001d041710 | Glutathione synthetase chloroplastic | 3 |
| 56 | Zm00001d041774 | Zea mays ARGOS9 | 2 |
| 57 | Zm00001d041880 | Beta-galactosidase 9 | 2 |
| 58 | Zm00001d043660 | PIN-formed protein8 | 2 |
| 59 | Zm00001d043923 | PAN domain-containing protein | 2 |
| 60 | Zm00001d044131 | Hypothetical protein | 2 |
| 61 | Zm00001d044767 | Hypothetical protein | 2 |
| 62 | Zm00001d045296 | DUF1674 family protein | 2 |
| 63 | Zm00001d045298 | Lipase-like | 2 |
| 64 | Zm00001d047074 | Pleckstrin homology domain-containing protein | 3 |
| 65 | Zm00001d047687 | Chaperone DnaJ-domain superfamily protein | 3 |
| 66 | Zm00001d047690 | Nucleic acid-binding proteins superfamily | 3 |
| 67 | Zm00001d047837 | Protein DA1-related 2 | 2 |
| 68 | Zm00001d048207 | RING zinc finger domain superfamily protein | 2 |
| 69 | Zm00001d048957 | Hypothetical protein | 2 |
| 70 | Zm00001d049179 | O-methyltransferase ZRP4 | 2 |
| 71 | Zm00001d049355 | Hypothetical protein | 3 |
| 72 | Zm00001d051362 | Tonoplast intrinsic protein2 | 2 |
| 73 | Zm00001d051851 | Plant/F8L15-130 protein | 3 |
| 74 | Zm00001d052033 | OSJNBb0045P24.5 protein | 2 |
| 75 | Zm00001d052372 | Protein LURP-one-related 8 | 2 |
| 76 | Zm00001d052605 | OVATE-transcription factor 21 | 2 |
| 77 | Zm00001d053416 | Inactive dual specificity protein phosphatase-like | 2 |
| 78 | Zm00001d053675 | lipoxygenase10 | 2 |
| 79 | Zm00001d053740 | Uncharacterised conserved protein UCP015417 | 2 |

**Supplemental Table 3**. Primer sequences used in this study

| **Primer name** | **Gene name** | **Gene Model** | **Forward primer sequence (5'-3')** | **Reverse primer sequence (5'-3')** | **Purpose** |
| --- | --- | --- | --- | --- | --- |
| Trans-F/R |  |  | AGCAGTACCTGGACGAGAACA | AGAAGTCGTGCTGCTTCATGT | Transgenic confirmation |
| S1-F/R |  |  | AGGAGTGCGAACGGTGAGT | TAGCAGGCTTGTTTGCTTGA | Mutant confirmation |
| GIF1-F/R | *gif1* | Zm00001d033905 | ATGCAGCAGCAACACCTGAT | ACTGCCTTCTTCCTCGGTGC | Coding sequence amplification |
| Q-gif1 | *gif1* | Zm00001d033905 | GCAGGTAGCTTTGAGGAACG | CATGCATGCTGGTCACTACC | RT-qPCR |
| Q-actin | *actin1* | Zm00001d010159 | TACGAGATGCCTGATGGTCAGGTCA | TGGAGTTGTACGTGGCCTCATGGAC |  |
| Q-an1 | *an1* | Zm00001d032961 | GGATGTTGCAAAAGGCAAAT | TGACACAGCCTCACCAATTC |  |
| Q-bd1 | *bd1* | Zm00001d022488 | GTACAACTACCCGCCCTTCC | GAGCCCTGGTGGTGGTAGT |  |
| Q-fea4 | *fea4* | Zm00001d037317 | CGGACATGACAACTCATTGC | GAAGCTCCTGCTCAAGATGG |  |
| Q-gt1 | *gt1* | Zm00001d028129 | CTTCAACTGATCCACGAGCA | CGGTCCATCCATCCATTAAC |  |
| Q-ra2 | *ra2* | Zm00001d039694 | CTTACGCTTCCGTGGCTAAT | GTCTCCCCCTTCCTTGTGAT |  |
| Q-rs1 | *rs1* | Zm00001d028129 | GGTGCAAGGACATTGTTGTG | CCCAGGATTCAAGTTCCAGA |  |
| Q-rte3 | *rte3* | Zm00001d028129 | AAACGCGTCTGTTGGTAAGG | CATTCCCCCTTTCATATCCA |  |
| Q-si1 | *si1* | Zm00001d036425 | TTAGCTACCGCGTGTGTACG | CATGGATGCAGATGAACAGG |  |
| Q-sid1 | *sid1* | Zm00001d019230 | CACAGGGGAAGGTGATGTCT | CGGTCCTCCTGTAGAAGGTG |  |
| Q-te1 | *te1* | Zm00001d042445 | AACATGCTGGACAACCACTG | AGCCGTAGCCCACATTACAC |  |
| Q-tpl2 | *tpl2* | Zm00001d028481 | CGTTCATCCTGTTGTGGTTG | CCATTTTCTCTCCGATTCCA |  |
| Q-ts6 | *ts6* | Zm00001d034629 | GCTGGTCTCCCTCTGGTATG | ACGCATGGTCGACTACAACA |  |
| Q-tsh1 | *tsh1* | Zm00001d039113 | CTCGCTTTAAAACCCAGCAC | GCGTATTGATGAGCCTTGGT |  |
| Q-tsh4 | *tsh4* | Zm00001d020941 | GAGCACTTTTGCATTGGACA | ACACGACGATCGATCAAATG |  |
| Q-ub2 | *ub2* | Zm00001d031451 | GGAGGCTGAAAGTCTGATGC | AGTCCCCGACAAGCATACTG |  |
| Q-ub3 | *ub3* | Zm00001d052890 | TCGCTCTAGGAGGCCAAATA | CGAGTTCAAACCATGGAGGT |  |

Supplemental table 3 (*continued*)

| Q-vt2 | *vt2* | Zm00001d008700 | CTCTCCTCCCTGCACTGAAC | GCATGCTCACACTGTTCCAC | RT-qPCR |
| --- | --- | --- | --- | --- | --- |
| Q-zfl1 | *zfl1* | Zm00001d002449 | AAGAACGGGCTCGACTACCT | GAACACCTGGTTGGTCACCT |  |
| Q-zfl2 | *zfl2* | Zm00001d026231 | TTCGTCTGCCTCTCATGTTG | AACTAACAAGTGGCCCATCG |  |
| Q-sk1 | *sk1* | Zm00001d002970 | GGACGAGATCACGAGAGACC | TTGCTAGCACGGATCACAAC |  |
| Q-tru1 | *tru1* | Zm00001d042111 | TGGCCCTCTATCATCCTCTG | GGCAACTCACCAAGATCCAT |  |
| Q-ts1 | *ts1* | Zm00001d003533 | GGGTGGGGAGAAATCTAAGC | CACCCATTCAGTTCCTCACA |  |
| Q-tls1 | *tls1* | Zm00001d032461 | AAATAAGCCCCGTTTTGCTT | GGCTTTTATTCGGACACGAC |  |
| Q-chip-UBI | *UBI* | Zm00001d015327 | GACGAGTCTAACGGACACCA | ATTTCTGGATGCCGACAGCG | ChIP-qPCR |
| Q-chip-cle4a | *cle4a* | Zm00001d034507 | TCCTTCTGGTGTCGTTCGTCGTG | TTGCTGTCCTGGAAGGGATC |  |
| Q-chip-opf21 | *opf21* | Zm00001d052605 | TCCGCCACCACGACCACAATG | CCGCATCGCTCATCCGTGGT |  |
| Q-chip-ago108 | *ago108* | Zm00001d013542 | TGATCATTGTGCCAAAGAATTT | CGATCCAGGAAAAGAACAAA |  |
| Q-chip-trps2 | *trps2* | Zm00001d028267 | CTCCTTCATCGACACAAGCA | GATCTCCGCATCTTCTTTGC |  |
| Q-chip-pin8 | *pin8* | Zm00001d043660 | CGCTGCCAAATACCTTGATT | AGCCCGAAACTCAAACAGAA |  |
| Q-chip-ra2 | *ra2* | Zm00001d039694 | CTTACGCTTCCGTGGCTAAT | GTCTCCCCCTTCCTTGTGAT |  |
| nLUC-gif1 | *GIF1* | Zm00001d033905 | CGAGCTCGGTACCCGGGATCCATGCAGCAGCAACACCTGAT | CGCGTACGAGATCTGGTCGACACTGCCTTCTTCCTCGGTGC | LCI assay |
| cLUC-Abp1 | *Auxin-binding protein 1* | Zm00001d041711 | TACGCGTCCCGGGGCGGTACCATGGCGCCGGATCTAAGCGA | ACGAAAGCTCTGCAGGTCGACTCACATCTTAGCAGGCGGTCTCG |  |
| cLUC-GRF1 | *GRF1* | Zm00001d017742 | TACGCGTCCCGGGGCGGTACCATGGCGATGCCGTATGCCTC | ACGAAAGCTCTGCAGGTCGACTTAGTCATCGTTGGGCGACTGGG |  |
| cLUC-GRF17 | *GRF17* | Zm00001d051456 | TACGCGTCCCGGGGCGGTACCATGGCCGAGGACAAGGAGAC | ACGAAAGCTCTGCAGGTCGACTCACGTGGCACGCGCGGCGCCGT |  |
| cLUC-NA2 | *nana plant2* | Zm00001d014887 | TACGCGTCCCGGGGCGGTACCATGGCGGACGTGCACGAACC | ACGAAAGCTCTGCAGGTCGACTTAGGCCTCCTCGTCCGCGTAGG |  |
|  |  |  |  |  |  |

References

1. Nelissen H, Eeckhout D, Demuynck K, Persiau G, Walton A, van Bel M, Vervoort M, Candaele J, De Block J, Aesaert S, Van Lijsebettens M, Goormachtig S, Vandepoele K, Van Leene J, Muszynski M, Gevaert K, Inzé D, De Jaeger G (2015) Dynamic changes in ANGUSTIFOLIA3 complex composition reveal a growth regulatory mechanism in the maize leaf. *Plant Cell* **27**: 1605–1619.
2. Vercruyssen L, Verkest A, Gonzalez N, Heyndrickx KS, Eeckhout D, Han SK, Jégu T, Archacki R, Van Leene J, Andriankaja M, De Bodt S, Abeel T, Coppens F, Dhondt S, De Milde L, Vermeersch M, Maleux K, Gevaert K, Jerzmanowski A, Benhamed M, Wagner D, Vandepoele K, De Jaeger G, Inzé D (2014) ANGUSTIFOLIA3 binds to SWI/SNF chromatin remodeling complexes to regulate transcription during Arabidopsis leaf development. *Plant Cell* **26(1):**210–229.
